# Interfacial Engineering of Dual‐Layer Au‐Shelled Ag Nanowires: Decoupling Chemical Stability From Ultrasensitive Surface‐Enhanced Raman Spectroscopy Activity

**DOI:** 10.1002/smsc.70401

**Published:** 2026-09-16

**Authors:** Yuyang Li, Ario Jafari, Dongqing Lin, Justinas Palisaitis, Yangpeiqi Yi, Jonas Oshaug Pedersen, Yulong Duan, Per O. Å. Persson, Magnus P. Jonsson, Thomas Ederth, Klas Tybrandt

**Affiliations:** ^1^ Laboratory of Organic Electronics Department of Science and Technology Linköping University Norrköping Sweden; ^2^ Division of Biophysics and Bioengineering Department of Physics, Chemistry and Biology Linköping University Linköping Sweden; ^3^ Division of Thin Film Physics Department of Physics, Chemistry and Biology Linköping University Linköping Sweden; ^4^ Wallenberg Initiative Materials Science for Sustainability Department of Science and Technology Linköping University Norrköping Sweden

**Keywords:** gold/silver nanowires, hierarchical, microplastics, surface‐enhanced Raman spectroscopy, stretchable electronics

## Abstract

One‐dimensional (1D) silver nanowires are promising surface‐enhanced Raman spectroscopy (SERS) substrates, yet their plasmonic hotspots are spatially confined to sharp tips or junctions, leaving the extensive body optically inactive. Additionally, realizing high‐performance Ag‐based substrates faces a fundamental trade‐off between chemical stability and surface activity. To simultaneously resolve these geometric and chemical limitations, we present a stepwise interfacial engineering strategy to synthesize 3D hierarchical dual‐layer porous@smooth Au‐shelled Ag nanowires. This architecture decouples functional requirements: A hermetic smooth Au inner shell passivates the Ag core for long‐term stability, while the nanowire body serves as a structural scaffold for a porous Au outer shell. This design activates the entire 1D surface, creating a volumetric distribution of high‐density hotspots localized at the interparticle gaps. Consequently, the hierarchical nanowires exhibit exceptional sensitivity for model analytes, including malachite green, crystal violet, and methylene blue, with detection limits ranging from 10^−11^ down to 10^−14^ M. Notably, for malachite green the sensitivity approaches the single‐molecule regime. Finally, leveraging their high aspect ratio and mechanical flexibility, the nanowires demonstrate versatile utility in two distinct applications: enabling both stretchable SERS substrates for conformal sensing and colloidal dispersions for the rapid “mix‐and‐measure” fingerprinting of microplastics (PE, PP, PS, and PET).

## Graphical abstract

A “Protection‐then‐Activation” strategy synthesizes 3D‐hierarchical AuNP‐Au@AgNWs, decoupling stability and surface‐enhanced Raman spectroscopy (SERS) activity. A hermetic inner Au shell protects the Ag core from corrosion, while overgrown porous Au nanoclusters generate dense volumetric hotspots. This robust 3D scaffold enables highly sensitive, label‐free SERS detection of dyes and microplastics on both rigid glass and flexible PDMS substrates.

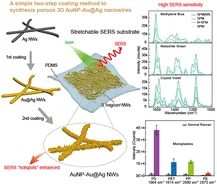

## 1 Introduction

Surface‐enhanced Raman spectroscopy (SERS) has emerged as a powerful analytical tool for chemical and bioanalytical sensing applications by exploiting localized surface plasmon resonance (LSPR) in metallic nanostructures [1, 2, 3]. One‐dimensional (1D) gold nanomaterials such as nanowires (NWs) are of particular interest as SERS substrates. In addition to excellent biocompatibility, chemical stability, and thermal robustness, their anisotropic geometry offers both longitudinal and transverse plasmon modes [4, 5, 6]. The longitudinal resonance is highly tunable and promotes intense electromagnetic fields, especially at sub‐10 nm nanogaps at ends, bends, and junctions. These regions act as effective “hotspots” due to pronounced charge accumulation [3, 7]. Beyond these individual features, plasmonic coupling between adjacent NWs creates extended hotspot regions at the interwire gaps, offering advantages for large‐area and uniform SERS sensing [8]. Additionally, the high aspect ratio of NWs enables the formation of interlocked NW meshes with excellent electrical conductivity, suitable for flexible and stretchable SERS substrates integrated into wearable devices [9, 10]. However, maximizing the potential of 1D substrates is hindered by a critical geometric limitation: In conventional smooth NWs, the hotspots are spatially localized at the sharp tips or interwire junctions, leaving the extensive surface area along the NW body optically “silent” or inactive [11].

Fabricating 1D Au‐based nanomaterials with both high hotspot density and structural robustness remains a significant synthetic challenge. The conventional bottom‐up seed growth method, while widely used for gold nanoparticles and nanorods, is often ineffective in synthesizing homogeneous 1D Au NWs exceeding 5 μm in length [12]. Rigid template synthetic routes, such as epitaxial growth of vertically aligned Au NWs on Si/SiO_2_ substrates [13, 14] or using sacrificial track‐etched membranes for thin‐walled Au nanotubes [15], enable important demonstrations but are constrained by complex processing, limited throughput, and surface morphology variability. Alternatively, surface‐suppressed galvanic replacement offers a scalable solution‐based method to form high‐aspect ratio bimetallic Au‐coated Ag NWs [16]. However, these methods typically produce smooth‐surfaced 1D gold nanostructures, whose enhancement is largely confined to tips or narrow interparticle gaps [17]. Surface roughening strategies can increase hotspot density. Controlled chemical etching on Ag NWs generates bead‐like surface features that increase the average enhancement factor by approximately 11‐fold [18]. Nanoparticle decoration via surface adsorption can form Au nanorods/carbon nanotube hybrids, which show a strongly enhanced Raman signal in the detection of Crystal Violet compared to dispersed Au nanorods or nanoparticles [19]. Another strategy involves the formation of mesoporous Au nanotubes by Au displacement of sputtered coatings or NWs [20, 21]. Still, they often compromise the hermetic seal of the Au shell, exposing the unstable Ag core to oxidation and galvanic corrosion. Thus, current strategies face a fundamental trade‐off between ensuring chemical stability (via smooth shells) and maximizing surface activity (via rough shells).

This trade‐off is particularly problematic when applying SERS to real‐world solid pollutants, such as microplastics (MPs). MPs have emerged as one of the most pervasive and insidious environmental challenges, due to their abundance and toxic effects [22]. SERS is a powerful technique for MP detection due to its high sensitivity and molecular specificity [23]. SERS enables detection of low concentrations and small MP fragments, often below the detection limits of conventional FTIR or pyrolysis‐gas chromatography/mass spectrometry (Py‐GC/MS) [24]. In addition, SERS enables rapid and potentially in situ analysis with minimal sample preparation, as well as identification of distinct molecular fingerprint information [25]. However, challenges remain in the development of more sensitive and specific assays for MPs' detection and identification, and particularly rigid or chemically unstable substrates often fail to provide either the conformational adaptability or the robust signal consistency required for in situ MPs fingerprinting.

In this work, we resolve these synthetic and analytical challenges via a “Protection‐then‐Activation” interfacial engineering strategy to synthesize 3D‐hierarchical Au nanoparticle‐coated Au/Ag core–shell NWs (3D‐AuNP‐Au@AgNWs) as high‐sensitivity SERS substrates. Unlike previous approaches, our strategy strictly decouples the functional requirements: We first grow an epitaxial, hermetic smooth Au inner shell to passivate the Ag core against corrosion, solving the stability issue. Subsequently, we orchestrate the kinetically controlled overgrowth of porous Au nanoclusters on this intermediate layer. In this architecture, the smooth Au‐coated Ag NW serves as a robust high‐aspect‐ratio template, supporting the dense loading of SERS‐active Au nanoparticles clusters. This hierarchical design transforms the optically inert NW body into a continuous “3D active scaffold”, creating a volumetric distribution of hotspots localized at the inter‐particle gaps. Consequently, this engineered architecture translates into exceptional label‐free SERS activity; limits of detection were determined for the dyes malachite green (MG), crystal violet (CV) and methylene blue (MB) on 3D‐AuNP‐Au@AgNW meshes on both glass and flexible/stretchable polydimethylsiloxane (PDMS) substrates. The porous NWs were also tested for their capacity to detect and distinguish four types of MPs, including polyethylene (PE), polypropylene (PP), polystyrene (PS), and polyethylene terephthalate (PET), at concentrations as low as 10 µg/mL for particles of 2 µm in average size. The combination of fast and scalable synthesis and high SERS sensitivity on both glass and flexible/stretchable substrates makes 3D‐AuNP‐Au@AgNWs an attractive platform for a wide range of SERS applications.

## 2 Results and Discussion

### 2.1 Synthesis and Characterization of Porous Au@Ag NWs

**FIGURE 1:**
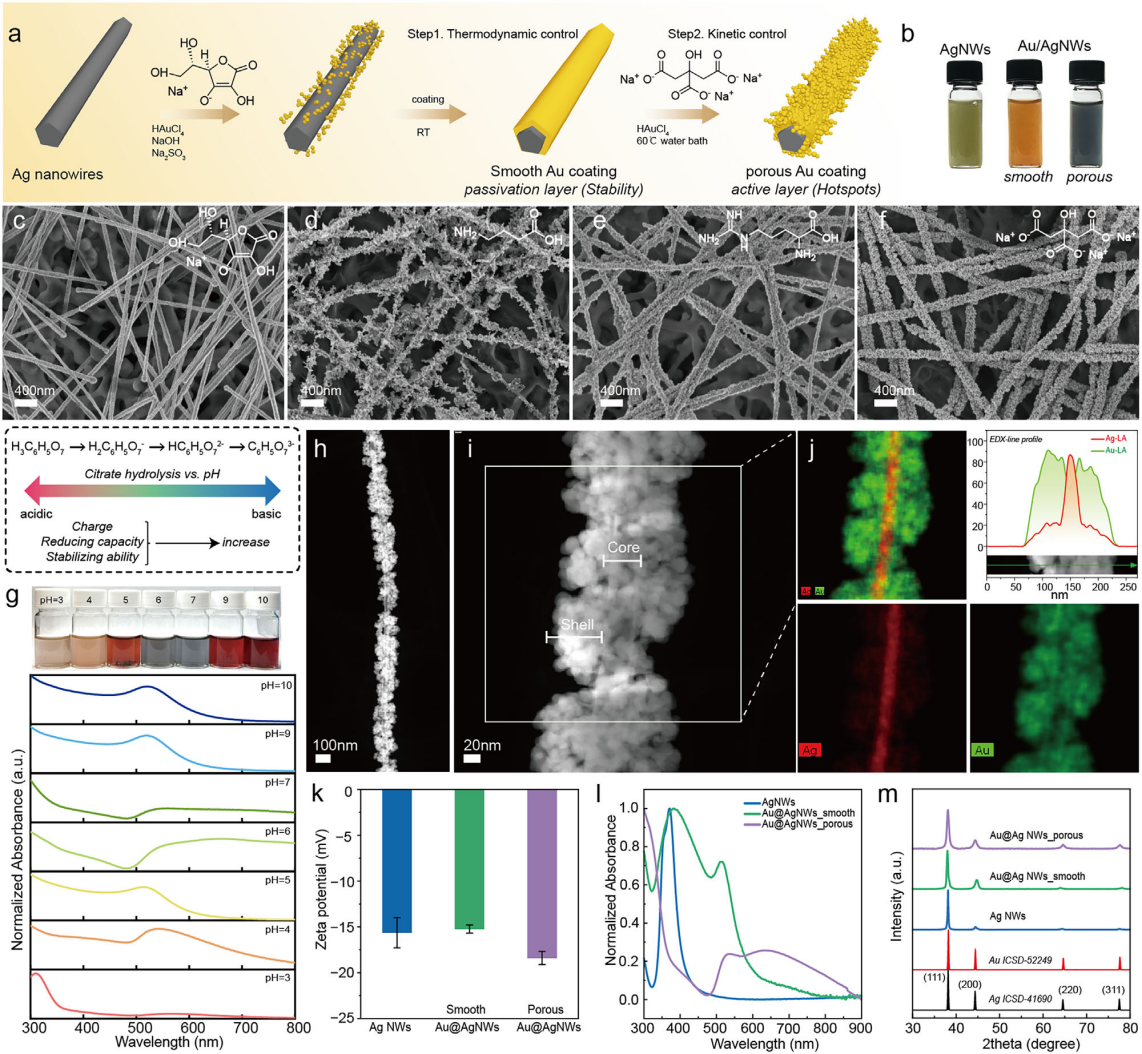
Synthesis and characterization of porous NWs. (a) Schematic of the two‐step synthesis routes of 3D‐hierarchical Au nanoparticle‐coated Au‐Ag core–shell NWs (3D‐AuNP‐Au@AgNWs). (b) Photos of dispersed silver NWs, first step synthesized smooth Au‐coated Ag NWs and second step synthesized porous Au coated Ag NWs. (c–f) Scanning electron microscope (SEM) imaging of second step Au‐coated Ag NWs synthesized with various reduction/capping agents: (c) sodium ascorbate, (d) gamma‐aminobutyric acid (GABA), (e) L‐arginine (Arg), and (f) sodium citrate. (g) UV–vis absorption spectra of 3D‐AuNP‐Au@AgNWs synthesized by the addition of sodium citrate for pH from 3 to 10. (h,i) High‐angle annular dark‐field–scanning transmission electron microscope imaging (HAADF–STEM) of porous 3D‐AuNP‐Au@AgNWs. (j) Energy dispersive X‐ray (EDX) elemental mapping (green box area) shows the Ag core and porous Au nanostructure coating, with an inset showing EDX‐line profiles of Ag and Au along the cross‐section. (k) Zeta potentials of Ag NWs, smooth Au@Ag NWs and porous 3D‐AuNP‐Au@AgNWs. Error bars represent mean ± SD for three repeated measurements per sample. (l) UV–vis absorption spectra of Ag NWs, smooth Au@Ag NWs, and porous 3D‐AuNP‐Au@AgNWs. (m) X‐ray powder diffraction (XRD) spectra of Ag NWs, smooth Au@Ag NWs, and porous 3D‐AuNP‐Au@AgNWs, with standard XRD spectra of Au ICSD‐52249 and Ag ICSD‐41690.

The porous 3D‐AuNP‐Au@AgNWs were synthesized by a stepwise interfacial engineering strategy, as shown in Figure 1a. In the first step, a smooth conformal Au layer was grown on commercially available Ag NWs (12 µm in length and 20 nm in diameter) dispersed in water. A gold sulfite solution composed of HAuCl_4_, NaOH, and Na_2_SO_3_ was used under basic conditions to suppress galvanic replacement of Ag [16]. Due to the high stability of the gold–sulfite complex, the release of gold ions is slow, favoring a thermodynamically controlled layer‐by‐layer epitaxial growth. This creates a dense, hermetic seal that suppresses the galvanic replacement of Ag and avoids etching and structural decomposition of the thin 1D AgNWs. A low concentration of ascorbate was used to reduce the Au complex, thereby forming a continuous smooth Au coating on the AgNWs, as shown in Figure S6b. In the second step, different additives, including sodium ascorbate (Asc), sodium citrate (Cit), L‐arginine (Arg), and gamma‐aminobutyric acid (GABA) were investigated to orchestrate the kinetically controlled overgrowth of porous Au coatings (Figure 1c–f). For sodium ascorbate, the color of the final solution was purple‐red, which is a sign of large amounts of free AuNPs [26]. The SEM image showed no significant growth on the NWs (Figure 1c), indicating that the gold seeds generated by the strong reducing agent ascorbate grew into NPs rather than depositing onto the body of the NWs [27]. There are a few reports of using GABA as a stabilizing and reducing agent through its amino and carboxyl groups, resulting in worm‐like Au nanostructures [28]. However, upon the addition of GABA, the solution color turned dark with clear aggregates, and SEM imaging showed uneven AuNP coating around the NWs (Figure 1d), indicating inhomogeneous growth and poor stabilization of the dispersion by GABA. Arg has similar functional groups as GABA, and the addition of Arg during the synthesis made the solution turn dark blue with clear aggregates. SEM images showed compact growth of Au nanoaggregates on top of the NWs, indicating weak stabilizing capacity and preferential seed growth onto the NWs. This is consistent with a previous report, where Arg induces coagulation during Au nanoparticle synthesis and results in different morphologies and large clusters [29]. For sodium citrate, the solution color turned dark gray, and SEM imaging revealed a homogeneous 3D AuNP coating around the NWs (Figure 1f). Citrate is known to be a milder reducing agent and an efficient stabilizer during colloidal AuNP synthesis through its carboxylate and hydroxy groups [30]. To explore the reaction conditions, different pH values were tested during the synthesis with Cit, resulting in differently colored samples (Figure 1g). At pH 6 and 7, the solutions showed a gray color and broad UV–vis absorption; the reduction kinetics favored heterogeneous nucleation while restricting surface diffusion. This induces a transition to the Volmer–Weber (island growth) mode, leading to the rapid accumulation of Au nuclei into hierarchical clusters on the NWs. At basic conditions, the wine‐red solution and the narrow absorption peak indicate dispersed AuNPs. At acidic conditions, the solution color and absorption spectra indicate smoother NWs, similar to the smooth Au@AgNWs. At pH 5, there were clearly sedimented NW aggregates at the bottom, and the solution color indicated dispersed AuNPs. The UV–vis absorption spectra in Figure 1g indicate the structural differences between the synthesis products at different pH values, with only the gray‐colored samples synthesized at pH = 6 and 7 yielding broad absorption peaks at 500–700 nm. This optimal condition balances the dispersion stabilization with the kinetic requirement for island growth. The wine‐red samples at pH = 5, 9, and 10 show similar relatively sharp peaks around 520 nm, while the gold‐colored samples at pH = 3 and 4 both show a broad peak around 550 nm consistent with smooth NWs. Controlling the pH is thus crucial to achieve growth of attached AuNPs onto the NWs, without NW agglomeration, to yield the porous morphology. The ionization state of Cit is highly affected by pH as indicated in Figure 1g, and the reductive capacity and stabilizing ability are optimal at pH around 6–7 for the porous gold nanoparticle coating process [31]. For pH below 5, the reductive capability of Cit is suppressed, and no major growth is observed. At pH = 5, gold is reduced, but the NWs are not sufficiently stabilized and agglomerate. At pH = 6–7, the NWs are well stabilized to remain in dispersion, but at the same time, the generated NPs attach to the NWs. For pH above 7, the purple solution color indicates that Au NPs are generated, but they are stabilized as discrete particles in solution instead of growing onto the NW templates, likely due to fast nucleation as citrate acts as a strong reductant at basic pH. The optimal condition thus balances the stabilization of the NW dispersion with limited stabilization of the generated AuNPs.

The structure of porous NWs was characterized by HAADF–STEM imaging (Figure 1h–j), revealing clusters of AuNPs attached all around the smooth Au@Ag NWs. The NW core had a diameter of ~70 nm, with adjacent clusters consisting of nanoparticles of approximately 20 nm in size. The elemental distribution was characterized by EDX spectroscopy elemental mapping, where a well‐defined Ag core coated with a thin smooth Au layer and a thicker porous Au layer is visible (Figure 1j). An EDX line profile, integrated along the length of the NW, confirms the core–shell structure (Figure 1j). The gaps between the particles are visible in the HAADF–STEM images. A comparison of UV–vis absorption spectra between AgNWs, smooth Au@AgNWs and porous Au@AgNWs is shown in Figure 1l. The pristine AgNWs show only a sharp peak at 390 nm, while the smooth Au coating broadens the peak above 400 nm and adds a second gold plasmonic peak at 520 nm. The porous Au coating created a broad peak above 600 nm, while the AgNW peak disappeared, likely due to etching by the HAuCl_4_. The broadening is an indication of aggregation or strong plasmon coupling and could be beneficial for SERS as it permits more flexibility in the selection of excitation wavelength (or reduced sensitivity to sample variations for a given wavelength) and potentially also facilitates resonance Raman or chemical enhancement upon molecular adsorption. The zeta potential was slightly more negative for the porous Au@Ag NWs (−18.4 mV ± 0.72) compared to the other NWs (Ag NWs, −15.6 mV ± 1.65; smooth Au@AgNWs, −15.2 mV ± 0.45) (Figure 1k), likely due to adsorbed citrate molecules. The negative zeta potential is also beneficial for detecting positively charged molecules by electrostatic attraction, resulting in enhanced SERS signals. The crystalline structure of the different NWs was investigated by XRD (Figure 1m). Although standard Au and Ag show indistinguishable spectra, preferential alignment of crystals can be seen as different ratios between the (111) and the (200) peaks [32]. The pristine AgNWs show the highest (111):(200) peak ratio, arising from the crystal orientation during nucleation and growth. The smooth Au@Ag NWs maintain some of the peak ratio, indicating that the crystal orientation is partially preserved. The porous Au@Ag NWs are more similar to the standard gold reference, likely due to the random orientation of the added gold NPs.

### 2.2 FDTD Simulations

**FIGURE 2:**
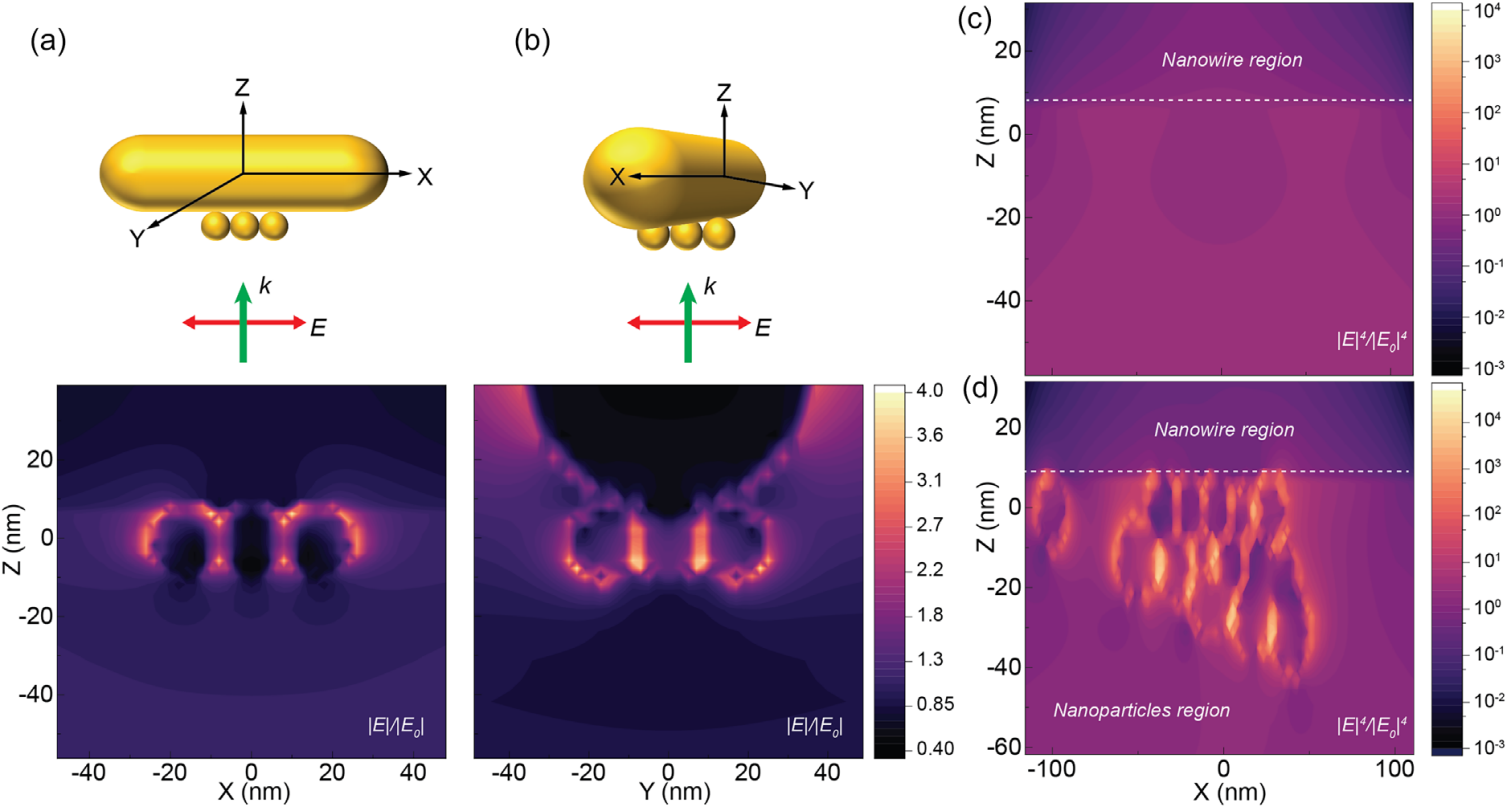
Finite‐difference time‐domain (FDTD) simulations of the plasmonic properties of smooth Au@AgNWs and porous 3D‐AuNP‐Au@AgNWs. (a,b) The basic models and near‐field distributions of a gold NW and three gold nanoparticles. In (a), the stacking direction of nanoparticles is along the *X*‐axis, parallel to the NW orientation, and the electrical polarization is also along the *X*‐axis. In (b) the stacking direction and the electrical polarization are along the *Y*‐axis. (c,d) Distributions of electromagnetic enhancement factors for pure NWs and NW‐nanoparticle aggregations, respectively. In both mappings, the electrical polarization was along the *X*‐axis and the wavelength was fixed near 532 nm.

To understand the optical effect of the NP coating, we investigated the near‐field distributions around NW‐nanoparticle aggregations through FDTD simulations. This model specifically isolates the geometric role of the NW as a structural scaffold. In Figure 2a,b, a NW was aligned along the *X*‐axis, and the light source was transmitted along the *Z*‐axis. Excitation with an electric field polarization (at a wavelength near 532 nm) parallel to the stacking direction of three closely spaced gold nanoparticles (with a uniform diameter of 16 nm) on the NW leads to clear hotspot regions driven by interparticle plasmonic coupling. Here, a small gap concentrates an intense electric field (with a large, normalized magnitude |*E*|/|*E*_0_| = 3–6) distributed along the edges or within the gaps (around 5 nm) of the nanoparticles (Figure 2a), where imperfections such as elliptical shapes, overlapping regions, and structural defects can influence |*E*|/|*E*_0_| (Figures S2–S4). Furthermore, the magnitude and distribution of these hotspot regions can be maintained even if the NW orientation is perpendicular to the stacking orientation of the nanoparticles (Figure 2b). These results suggest that the NW functions primarily as a 1D template supporting the nanoparticle shell, and its specific orientation does not significantly influence the surface plasmon resonances of the nanoparticle aggregates. On this basis, we further investigated the effect of gold nanoparticles on the electromagnetic enhancement factors of electric fields, quantified by |*E*|^4^/|*E*_0_|^4^ near the wavelength of 532 nm [33]. As shown in Figure 2c, the pure NW does not exhibit significant electromagnetic enhancement upon the radiation of the light with a wavelength of 532 nm. However, the incorporation of gold nanoparticles (with diameters ranging from 10 to 20 nm) near NWs generates a strong enhancement factor around the edges of the nanoparticles and the gaps between adjacent nanoparticles, effectively transforming the inert NW body into a 3D active volume. Around the hotspot regions generated by the semienclosure of three nanoparticle aggregates, the |*E*|^4^/|*E*_0_|^4^ ratio can increase by four orders of magnitude (Figures 2d and S5) compared to that at the outer sides of nanoparticle aggregates. Moreover, |*E*|^4^/|*E*_0_|^4^ sharply decreases to around 100 without the hotspot regions caused by the nanoparticles. Considering that organic molecules can be attached at the nanoparticle edges (in semienclosed configurations, Figure S5) overlapping with the hotspot regions, such a hierarchical scaffold has the potential to enable more efficient SERS performance in sensing applications.

### 2.3 SERS Performance of Porous Au@Ag NWs

**FIGURE 3:**
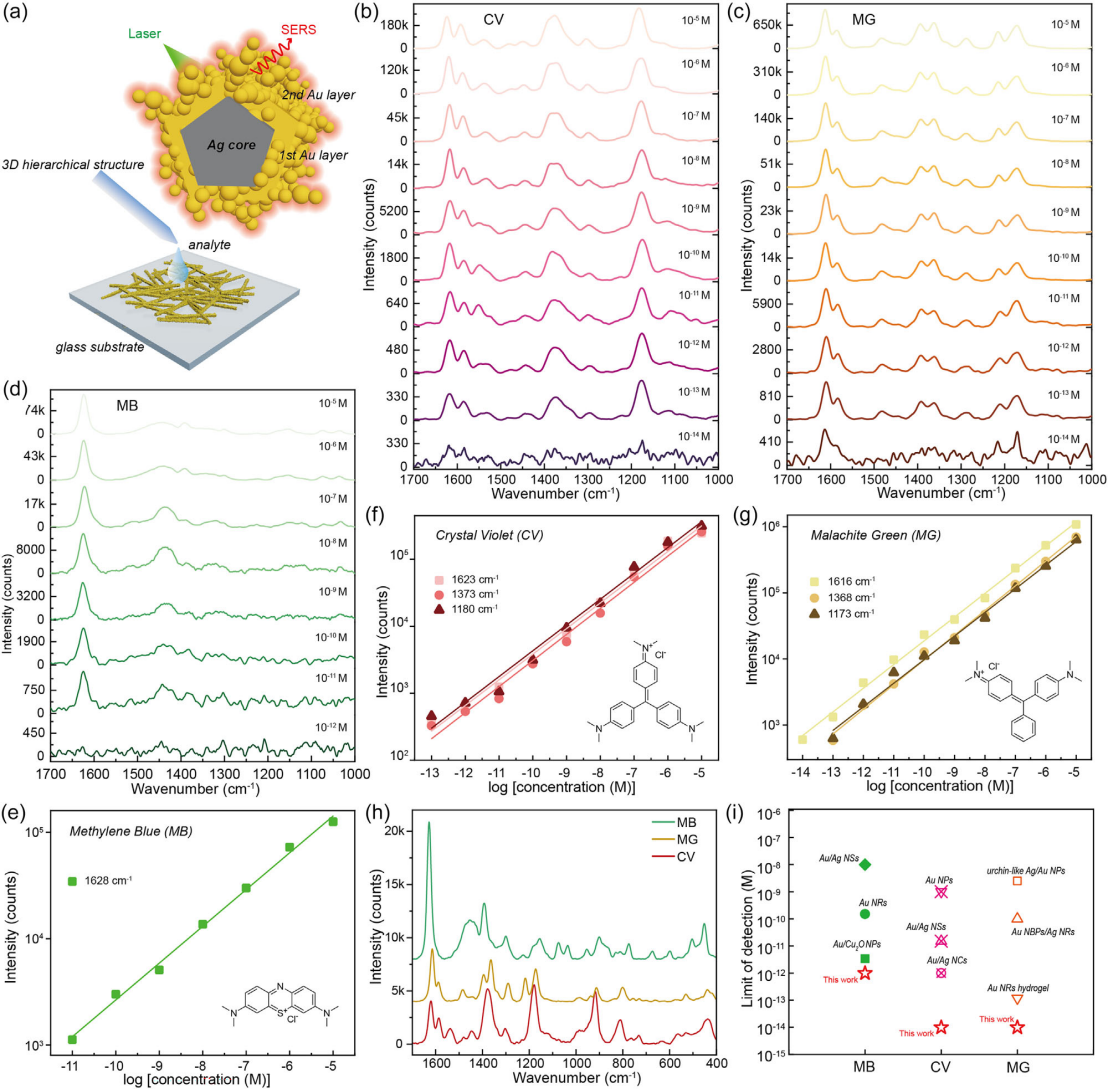
SERS sensitivity assessment of methylene blue (MB), malachite green (MG), and crystal violet (CV) molecules on porous 3D‐AuNP‐Au@AgNWs on glass substrates. (a) Schematic of the procedure. Raman spectra of different concentrations of (b) CV, (c) MG, and (d) MB on the NW SERS substrates (*λ*_0_ = 532 nm, 10 mW, 30 s). Note the individual intensity scales for each spectrum. The logarithmic changes in the intensities of the Raman maxima for selected peaks of each analyte, versus the concentration, for (e) MB, (f) CV, and (g) MG. (h) Normal Raman spectra of 10^−4^ M CV, MG, and MB (*λ*_0_ = 532 nm, 10 mW, 60 s). (i) Comparison of limits of detection of the three dye molecules by various Au‐based SERS substrates in the literature (see Table S3 for details and references). Error bars in panels e–g represent mean ± SD from 15 spectra per concentration. Detailed signal‐to‐noise ratio (SNR) statistics, including RSD, 95% CI, and *p*‐values, are provided in Table S5.

To characterize the SERS sensitivity of porous 3D‐AuNP‐Au@AgNWs, the common model analytes MB, CV, and MG were used to evaluate the performance. By directly drop‐casting analytes at different concentrations ranging from 1 × 10^−5^ to 1 × 10^−14^ M onto the SERS substrates, the obtained average spectra from 15 measurements (3 samples and 5 random points from each sample) are shown in Figure 3. The reference Raman spectra of the analytes are given in Figure 3h, and band assignments are summarized in Table S1. The overall SERS spectral intensity gradually decreases as the concentration of the analyte solutions decreases. Notably, for MG, distinct Raman fingerprints remain discernible down to 10^−14^ M. This femtomolar‐level sensitivity experimentally validates the hierarchical field enhancement predicted by our FDTD simulations (Section 2.2), confirming that the coupling between the NW body and the porous Au shell amplifies the electromagnetic field sufficiently to detect analytes approaching the single‐molecule regime.

To quantitatively validate the limit of detection (LOD), the SNR was calculated for the characteristic Raman bands of each analyte (MG: 1616 cm^−1^, CV: 1623 cm^−1^, and MB: 1628 cm^−1^). The SNR was defined as the ratio between the baseline‐corrected peak intensity and the standard deviation of the noise region. The results (Figure S17) show that MG exhibits detectable signals down to 10^−14^ M (SNR ≈ 3), whereas CV and MB reach reliable detection limits of 10^−13^ and 10^−11^ M, respectively. At lower concentrations, the signals approach the noise level and do not satisfy the SNR ≥ 3 criterion. To support this threshold more quantitatively, the SNR values were further evaluated using the mean SNR, standard deviation, relative standard deviation, 95% confidence interval, and a one‐sample Student's *t*‐test against the SNR = 3 criterion (Table S5). This analysis supports the reported detection limits for MG, CV, and MB while also showing the expected increase in variability close to the lowest detectable concentrations.

The spectral intensities vary among analytes, with MG being significantly stronger than CV and MB. These differences are partly a result of general characteristics of the analytes [34], but may vary depending on the interaction between a substrate's surface chemistry and the molecular properties of the analytes, including the orientation of the analyte molecules on the SERS surface, possible differences in chemical enhancement, and the charge transfer between the surface and the analyte molecules. As shown in Figure 1k, the porous Au shell has a negative zeta potential (≈−18 mV), which is favorable for attracting the cationic dyes MG, CV, and MB toward the NW surface. This electrostatic attraction can increase the local amount of dye close to the active surface and therefore helps improve the analytical response. However, this effect should be distinguished from the SERS enhancement mechanism itself. The strong Raman amplification mainly arises from the plasmonic hotspots created by the porous Au nanostructure, as supported by the FDTD simulations and the established SERS mechanism [1]. In addition, molecular orientation and possible charge–transfer interactions may influence the relative intensities of individual Raman bands [35]. It is worth noting that orientation can directly affect how close certain parts of the analyte molecules are to the metal surface, which in turn influences the level of electromagnetic enhancement. In contrast, chemical enhancement mechanisms—such as charge transfer interactions and changes in molecular polarizability—are considered separate from electromagnetic effects, as they result from electronic interactions with the surface rather than enhanced local fields [35].

Both CV and MG show similar trends in band‐intensity reduction as the analyte concentrations decrease. All characteristic bands remain visible at a concentration of 1 × 10^−13^ M for both analytes. However, at 1 × 10^−14^ M, the bands begin to fade into the BG noise, although some peaks are still discernible in the MG spectrum at this low concentration. For MB, the characteristic band remains reliably detectable down to 1 × 10^−11^ M based on the SNR ≥ 3 criterion, indicating a higher detection limit compared to CV and MG. The shapes of the characteristic bands corresponding to C—C ring stretching, C—N stretching, and C—H bending (1623, 1373, and 1180 cm^−1^ in CV, and 1616, 1368, and 1173 cm^−1^ in MG) remain consistent across all spectra, making them reliable indicators for analytical detection down to 1 × 10^−13^ M. The intensities of these peaks increase as a power function with rising CV and MG concentrations. The logarithm of the peak intensities shows a linear relationship with the logarithm of the analyte concentration, with coefficients of determination (*R* ^2^) averaging 0.985 for CV and 0.994 for MG, as shown in Figure 3f,g (the slight but systematic deviation from linearity, especially for CV in Figure 3f, is discussed below but is of minor relevance here). For MB, the characteristic C—C ring stretching band at 1628 cm^−1^ follows a similar behavior to the corresponding bands in CV and MG down to 1 × 10^−11^ M. The log–log linear relationship between MB concentration and peak intensity also shows a strong correlation, with a coefficient of determination (*R* ^2^) of 0.996, as shown in Figure 3e. However, the shape and position of the asymmetric (1454 cm^−1 ^→ 1438 cm^−1^) and symmetric (1392 cm^−1 ^→ 1379 cm^−1^) C—N stretching bands in MB show noticeable changes as the concentration decreases on the SERS substrate, compared to the bulk MB spectrum. This shift is likely due to charge transfer effects during the chemical enhancement process and interactions between the surface and the molecule. These changes reduce the accuracy and reliability of using these specific bands as analytical indicators. In comparison with previous work on Au nanomaterial‐based SERS substrates, 3D‐AuNP‐Au@AgNWs show better performance than Au/Ag bimetallic nanoparticles in the detection of CV and MG molecules and nearly equivalent performance for MB (Figure 3i). It should be noted that this work uses chemically synthesized NWs deposited on glass substrates, without postprocessing such as compositing additives like graphene or integrating the NWs into substrates such as carbon nanotubes or hydrogels to enhance the SERS properties.

### 2.4 Stretchable 3D‐AuNP‐Au@Ag NWs‐PDMS Substrate

**FIGURE 4:**
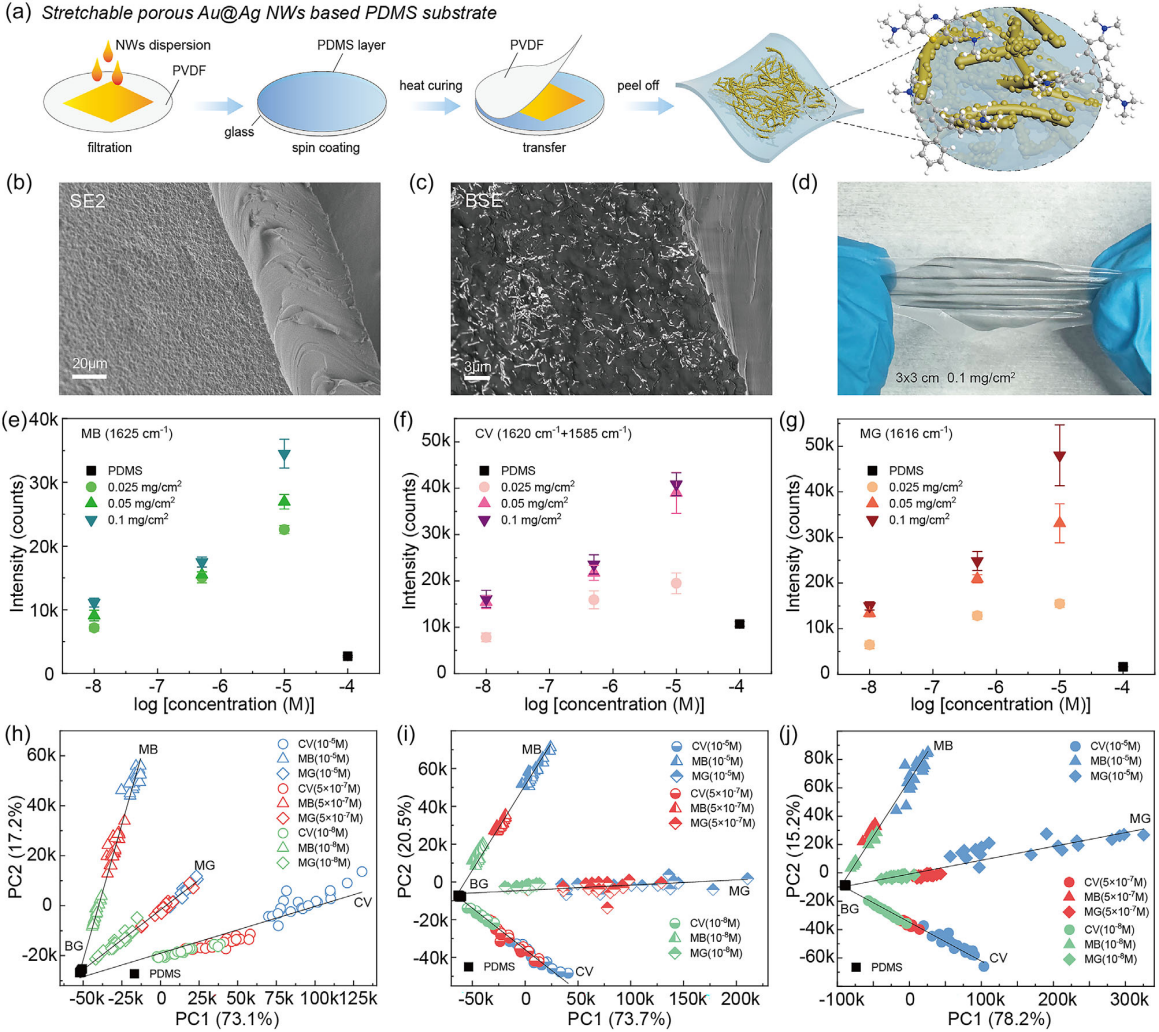
3D‐AuNP‐Au@AgNW‐PDMS stretchable SERS substrate. (a) Substrate fabrication route by vacuum filtration and transfer onto a PDMS layer. SEM imaging of 3D‐AuNP‐Au@AgNW‐PDMS substrate on a 45° inclined stage: (b) by secondary electron (SE2) imaging, (c) by backscattered electron (BSE) imaging, and (d) photo of flexible/stretchable substrate. (e–g) SERS peak maxima from stretchable 3D‐AuNP‐Au@AgNW‐PDMS substrates with added analytes (e) MB, (f) CV, and (g) MG on substrates with NW densities of 0.025, 0.05, and 0.1 mg/cm^2^ and at different analyte concentrations. Principal component analysis (PCA) score plots of the Raman spectra from various analyte concentrations on PDMS‐based SERS substrates with different NW loadings: (h) 0.025 mg/cm^2^, (i) 0.05 mg/cm^2^, and (j) 0.1 mg/cm^2^. BG points correspond to spectra collected from clean substrates prior to analyte deposition. Error bars in panels e–g represent mean ± SD from 20 spectra per concentration.

Stretchable SERS substrates can deform, bend, and fold onto a variety of geometrical forms, making them attractive for wearable electronics, in situ detection on curved surfaces, and portable analytical devices [10]. Here, we have developed a stretchable SERS substrate by transferring a mesh of porous Au@Ag NWs onto a soft and stretchable polydimethylsiloxane (PDMS) substrate (Figure 4a). The porous Au@Ag NWs were partially embedded into the PDMS surface, which anchors them robustly to the substrate (Figure 4b–d). This allows the SERS substrate to be stretched and adhered to curved surfaces (Figure S8).

PDMS substrates with different loadings of porous Au@Ag NWs (Figure S7) were used as SERS substrates to characterize the sensitivity. The Raman spectra of PDMS substrates with different NW loadings, measured without analytes, showed the expected characteristic PDMS peaks (Figure S11) [36]. For the same analyte concentration, spectra collected on the SERS glass substrates discussed above were more intense than on SERS PDMS substrates. On PDMS, even an angström‐thick polymer overlayer—or sinking and partial encapsulation—acts as a dielectric spacer that increases analyte‐hotspot separation, and, because SERS enhancement scales with |*E*|^4^, this gap can reduce the signal by orders of magnitude [37]. Additionally, the flatter glass interface offers slightly better optical coupling (cleaner) and fewer barriers to adsorption, and its higher refractive index than PDMS shifts the LSPR and near‐field distribution in ways that can support stronger localized fields for certain particle geometries [38]. Increasing the loading density of porous Au@Ag NWs on PDMS substrates led to higher SERS intensities for all analytes, consistent with a larger population of surface hotspots. The intensities of selected peaks for each analyte (CV [1620 and 1585 cm^−1^], MG [1616 cm^−1^], and MB [1625 cm^−1^]) were compared versus analyte concentrations on different substrates (Figure 4e–g, full spectra are shown in Figure S9).

To better understand this behavior and estimate the detection limits of the PDMS‐SERS substrates, we measured SERS spectra across a range of analyte concentrations using the highest Au@AgNW loading on PDMS (0.1 mg/cm^2^). The results (Figure S16a–c) show that CV, MG, and MB remain detectable down to 10^−11^, 10^−12^, and 10^−10^ M, respectively. These low detection limits indicate that PDMS‐SERS substrates remain competitive with glass substrates for practical sensing. By comparing the results from the PDMS and the glass substrates, the decrease in intensity with decreasing analyte concentration is qualitatively very different on PDMS (Figure S16d–f) as compared to glass (Figure 3e–g). For CV, the glass/PDMS intensity ratio for the summed bands at 1620 and 1585 cm^−1^ was 10.62 at 10^−5^ M, 2.15 at 10^−8^ M, and 1.16 at 10^−11^ M, respectively. The slower drop of band intensity on PDMS substrates comes from adsorption‐limited occupancy and site heterogeneity of the hotspots. At high concentrations, so many hotspot sites are occupied, and the glass signal is much larger (ratio for CV ≈10.6 at 10^−5^ M). As concentration decreases, the occupancy of hotspot sites on glass falls quickly, whereas the PDMS surface, having fewer hotspots, retains a small set of occupied sites at much lower analyte concentrations (≈2.15 at 10^−8^ M and ≈1.16 at 10^−11^ M). This behavior is naturally explained by adsorption‐limited occupancy of hotspots.

The concentration–intensity dependencies are well described by the Langmuir–Freundlich isotherm, which is derived from the Langmuir isotherm but is better suited for modeling heterogeneous surfaces [39]:

$$I\left(C\right)=b+I_{\max}\frac{{\left(KC\right)}^{n}}{1+{\left(KC\right)}^{n}}$$

where *b* is the BG intensity; *C* is the analyte concentration; *K* is the affinity constant; *I*_max_ is the maximum SERS intensity; and *n* is the heterogeneity index, which yields the Langmuir model for *n* = 1 and indicates heterogeneity for *n* < 1. This model reduces to a power law, *I* ∝ *C* ^*n*^, at low surface coverage and approaches saturation at high concentration. The log–log (intensity–concentration) plots for CV, MG, and MB are shown in Figure S16d–f, including fits to this model (the fit parameters are presented in Table S4). Results (Table S4) show that PDMS substrates exhibit larger *K* and a slightly larger *n* (i.e., a more Langmuir‐like shape), whereas glass has a much higher *I*_max_ despite its lower *K* and lower *n* (greater heterogeneity). A possible explanation is that hydrophobic dyes diffuse into PDMS, increasing the apparent affinity *K* but leaving fewer molecules at metal hotspots compared with glass substrates [40, 41]. By comparing the normal Raman spectrum of CV with the SERS spectra collected on PDMS‐supported NW substrates, a noticeable inversion in the relative intensity of the C—C ring stretch peaks at 1620 and 1585 cm^−1^ is observed (Figure S9), where 1620 cm^−1^ is stronger in normal Raman, while 1585 cm^−1^ is stronger in SERS. This trend is further quantified by the intensity‐ratio analysis presented in Figure S10. This behavior can be related to differences in the interactions of the molecules with the different SERS substrates, where vibrational modes may exhibit significantly different enhancement factors depending on the relative orientations of the vibrational dipole moments and the local electromagnetic fields [35]. To better understand the molecular discrimination and sensitivity performance, PCA was applied to the data obtained for the different SERS substrates, considering both the analytes and their concentrations (Figure 4h–j). It should be noted that the PCA analysis was performed on the PDMS‐supported NW platform to evaluate spectral discrimination behavior. In contrast, the lowest detection limits reported in this study were obtained using the nanoparticle‐coated NW substrate on glass, which provided stronger enhancement and higher sensitivity. Therefore, the PCA concentration range and the final LOD values correspond to different substrate configurations and should not be directly compared. The PCA score plots show clear clustering for each analyte and concentration, confirming the capability of the SERS substrates to resolve their molecular signatures based on characteristic vibrational profiles. For all datasets, the first two principal components (PCs) account for over 90% of the total variance, which indicates that the key spectral information is well preserved, allowing concentration‐dependent separation in a reduced two‐dimensional space. The results also confirm that SERS substrates with higher NW loading yield more distinct clustering and improved separation among analytes, which can be attributed to the increased density of plasmonic hotspots and enhanced reproducibility of the SERS effect. The loading plots (Figure S12) show that the major contributors to variance are associated with the C—C vibrational modes of the analytes. Overall, PCA provides a clear visual representation of concentration‐dependent sensitivity for the different analytes. Beyond the specific performance on PDMS, the success of this stretchable substrate highlights the fundamental mechanical adaptability of the 1D NW network. Unlike 0D nanoparticles that require rigid support, the high‐aspect‐ratio NWs form an interwoven mesh that can physically deform and conform to complex topographies. This same geometric advantage is the ability to wrap around and bridge irregular interfaces, not only limited to solid films but also serving as the key mechanism enabling the liquid‐phase detection of solid MP pollutants, as demonstrated in the following section.

### 2.5 SERS Detection of MPs

**FIGURE 5:**
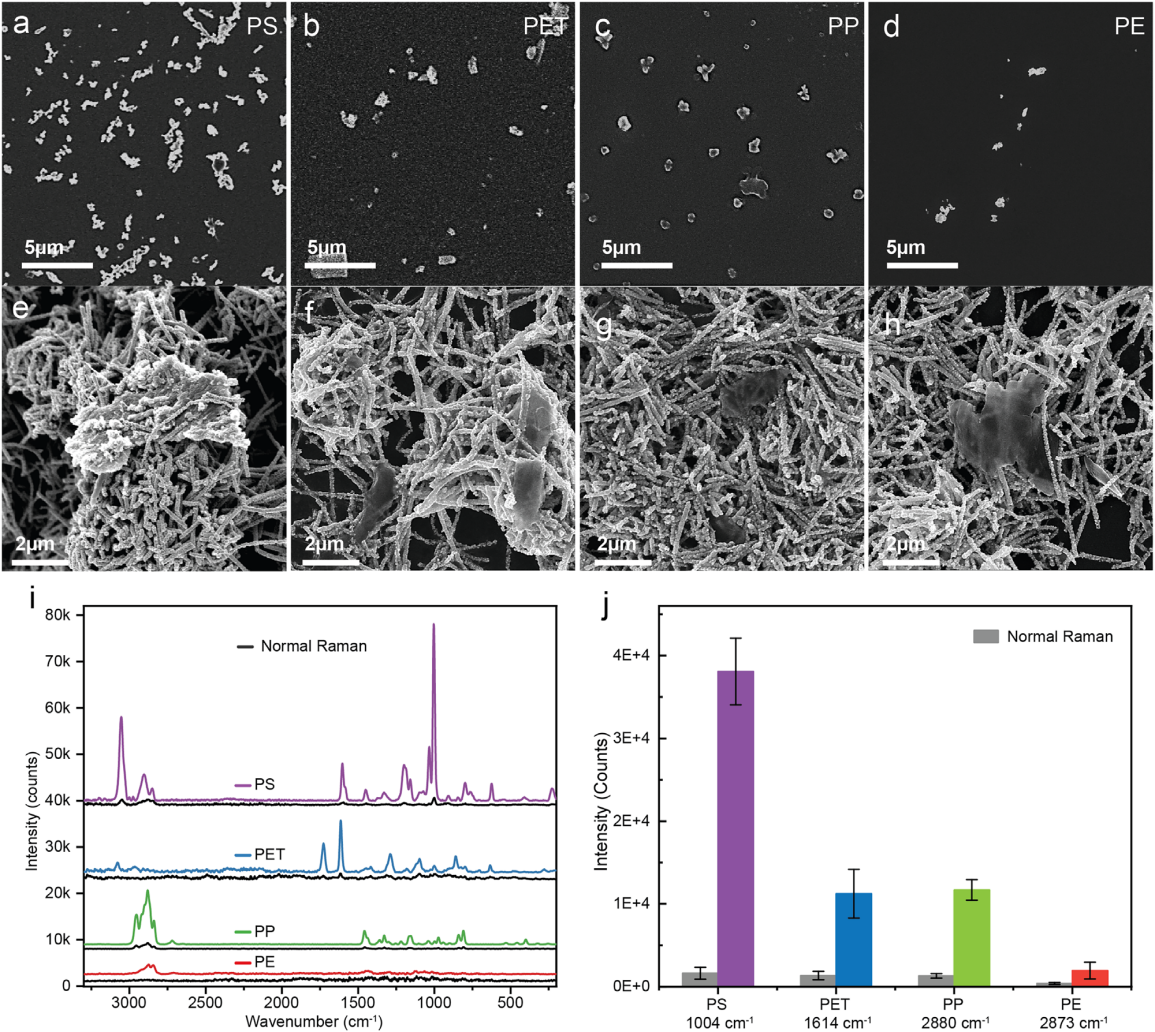
SERS detection of MPs of polyethylene (PE), polyethylene terephthalate (PET), polypropylene (PP), and polystyrene (PS). SEM images of (a) PS, (b) PET, (c) PP, and (d) PE MPs on glass substrates. SEM images of MPs mixed with 3D‐AuNP‐Au@AgNWs as SERS particles for (e) PS, (f) PET, (g) PP, and (h) PE). (i) Raman spectra of MPs (normal Raman) on the glass substrate compared with MPs mixed with Au@Ag NWs (SERS) on the glass substrates (*λ*_0_ = 532 nm, 10 mW, 60 s; spectra have been offset vertically for clarity). (j) Comparison of band intensities in normal Raman and SERS for each polymer. Error bars in panel j represent mean ± SD from 25 spectra per polymer. Detailed statistical values are provided in Table S6.

Beyond the ultrasensitive detection of dissolved small molecules, we further demonstrated the versatility of the 3D‐AuNP‐Au@AgNWs for the detection of solid environmental pollutants. Using a facile “mix‐and‐measure” detection strategy, the NW dispersion was mixed with MP particle suspensions, as shown by the SEM images. In Figure 5a–d, the MP particles were mostly smaller than 2 μm. PS MPs exhibited a higher degree of aggregation compared to those made from other plastics, and in some cases PS particles cluster into aggregates as large as tens of micrometers (see Figure S13). Although PP, PET, and PE MPs also showed some aggregation, the aggregates were composed of much fewer particles. The PS particles’ tendency to aggregate can be attributed to their higher hydrophobicity due to the presence of phenyl groups, as well as the fact that PS particles typically exhibit a lower zeta potential, reducing electrostatic repulsion [42].

Upon mixing with MP suspensions, the hierarchical NWs spontaneously adsorb onto the plastic surfaces, as shown in Figure 5e–h. This interaction is facilitated by the high‐aspect‐ratio 1D morphology of the NWs. Although their diameter is below 100 nm, their length is approximately 12 µm, which is larger than the typical MP particle size used here (~2 µm). Therefore, the NWs can form an interwoven plasmonic mesh around the MP particles and establish multiple close‐contact points through physical interactions. This close contact between the NW network and the plastic surface enables enhancement of the Raman fingerprints of the MPs without requiring complex substrate fabrication or precise particle alignment.

To quantitatively evaluate the SERS activity of the porous Au@Ag NWs for MP detection, samples for both normal Raman and SERS measurements were prepared. For each sample, five distinct areas were tested, and five spectra from each area were captured. The averages of these 25 spectra are presented in Figure 5i for each type of plastic. Unlike small‐molecule analytes, MPs are irregular solid particles with nonuniform local density, aggregation, orientation, and contact with the NW network. Therefore, these measurements demonstrate practical MP fingerprinting rather than strict spatial uniformity across a flat analyte layer. To further support the reproducibility of the MP measurements, the integrated areas of selected characteristic SERS bands were statistically evaluated using the mean value, standard deviation, relative standard deviation, 95% confidence interval, and Student's *t*‐test (Table S6). The results show acceptable reproducibility for PS and PET, moderate variability for PE, and higher variability for PP. This behavior is reasonable for MP samples, where particle size, local particle density, aggregation, orientation, and contact with the NW network can vary between measurement positions.

To provide a reliable evaluation of Raman fingerprint integrity, we extended the analysis beyond a single characteristic peak. After baseline correction, both normal Raman and SERS spectra were evaluated using locally integrated band areas within moving windows (Δ = 10 cm^−1^). This approach allowed us to examine how enhancement varies across different vibrational regions for each polymer. Results show that the degree of enhancement was not uniform across the spectrum. This variation can be attributed to differences in Raman scattering cross‐section, vibrational polarizability, and the interaction of specific modes with localized plasmonic hotspots. In addition, the orientation and contact between the polymer surface and the NW network can influence which bands are most strongly enhanced. The results also show consistent enhancement across multiple vibrational modes for all four polymers (PE, PP, PET, and PS), as represented in Table S7.

The results show that SERS samples yield enhanced peak intensities for all types of MPs compared with their normal Raman spectra. The degree of enhancement varies between the samples, with PS MPs showing the highest enhancement. The stronger enhancement for PS can be linked to its high polarizability due to the phenyl groups (aromatic rings) in its structure. These rings, with their delocalized electrons, can interact readily with the plasmonic surface and align more easily with hotspots, maximizing the SERS effect. In PET, the aromatic ring and ester linkage also contribute to spectral enhancement, though to a lesser extent than in PS. The presence of ester groups slightly reduces PET's hydrophobicity, which affects its ability to aggregate and align near hotspots, though PET still enhances more effectively than polyolefins such as PP and PE. PP and PE, on the other hand, have simple aliphatic structures, lacking aromatic rings, and exhibiting lower polarizability, which limits their SERS enhancement. PP has slightly higher hydrophobicity than PE due to the methyl group in its backbone, which may allow it to interact better with the SERS surface than PE. Estimated EF values for the bands compared in Figure S18 are 34 ± 4 for PS, 9.2 ± 0.3 for PET, 11 ± 1 for PP, and 9.7 ± 0.5 for PE, which are admittedly modest but still sufficient for reliable identification at 10 µg/mL. Here, the absolute signal intensity is influenced by the random nature of the adsorption compared with substrates using nanoparticles on lab‐prepared MPs [22, 43], patterned plasmonic surfaces [44], or melt‐assisted contact on model particles [44, 45]. Nevertheless, this method offers a decisive advantage in throughput and simplicity. By bypassing complex fabrication steps, the 1D NW network provides a robust, low‐cost tool for the rapid, in situ identification of environmental MPs, validating the practical robustness of our interfacial engineering strategy.

In real natural water samples, several factors can affect how the NWs interact with the MPs. Changes in pH and salt concentration can shift the surface charge and influence aggregation behavior [46]. Natural organic matter can also adsorb onto the plastic surface and form a coating, which may partially block direct contact with the NWs [47]. In addition, pigments or other contaminants present on environmental MPs can increase fluorescence BG and complicate Raman identification [48]. Therefore, the behavior observed here should be considered to occur under simplified conditions, and future tests in more complex matrices will be needed to evaluate practical performance.

## 3 Conclusions

In summary, we have successfully engineered a dual‐layer Au‐shelled Ag NW architecture that simultaneously achieves chemical robustness, volumetric hotspot generation, and mechanical adaptability, with capabilities that have remained mutually exclusive in conventional 1D plasmonic systems. This approach strictly decouples the functional requirements of the substrate: The hermetic smooth Au inner shell effectively passivates the Ag core to ensure long‐term chemical stability, while the NW body serves as a structural scaffold for the porous Au outer shell. This synergistic architecture activates the entire 1D surface by transforming the optically inert NW body into a continuous 3D active volume, creating a volumetric distribution of hotspots localized at the interparticle gaps. The effect is confirmed by FDTD simulations and by the experimentally demonstrated femtomolar SERS sensitivity for dye molecules. Furthermore, leveraging the unique high aspect ratio of the NWs, we demonstrated their versatile utility in two distinct applications: serving as both stretchable solid substrates for conformal sensing and as colloidal dispersions for the rapid “mix‐and‐measure” fingerprinting of MPs (PE, PP, PS, and PET) via physical entanglement. Overall, this work establishes a design principle for creating multifunctional 1D plasmonic nanostructures that are simultaneously chemically robust, optically ultrasensitive, and mechanically adaptive. This approach opens new pathways toward next‐generation SERS substrates, reconfigurable optical materials, and adaptive plasmonic systems for complex real‐world applications.

## 4 Experimental Section

### 4.1 Materials

Ag NWs with a diameter of 20 nm (±2 nm) and length 12 μm (±2 μm) (from Sigma–Aldrich) were dispersed in water (5 mg/mL). Gold (III) chloride solution (HAuCl_4_, 99.99% trace metals basis, 30 wt% in dilute HCl), sodium hydroxide (NaOH, 99.99% trace metals basis), sodium sulfite (Na_2_SO_3_, ≥98.0%), hydrochloric acid (HCl, 37%), polyvinylpyrrolidone (PVP, average *M*_W_ ~ 55 000), sodium citrate (Na_3_C_6_H_5_O_7_·2H_2_O, ≥99.0%), and sodium ascorbate (C_6_H_7_NaO_6_, ≥99.0%) were from Sigma–Aldrich. All chemicals were used as received.

### 4.2 Two‐Step Au‐Coating of Ag NWs

First, to prepare gold sulfite basic solution as the precursor, 26 µL HAuCl_4_ was diluted with 1.575 mL DI water, then 0.229 mL 1 M NaOH was added, and the mixture was heated for 15 min at 60 °C in a water bath. After cooling under tap water, 1.1 mL 0.1 M Na_2_SO_3_ was added, and the solution was left statically for 6 h. For smooth Au coating of the Ag NWs, 0.793 mL 5 mg/mL Ag NW solution was dispersed in 4.829 mL DI water, then 10.867 mL PVP 55k 5 wt% solution was added under stirring; thereafter, 0.194 mL 0.1 M Na_2_SO_3_ and 0.388 mL 1 M C_6_H_7_NaO_6_ were added subsequently. Then, 2.929 mL of the gold sulfite solution was added. After stirring for another 1 h, the solution turns totally brick red and goldish. Finally, the Au‐coated Ag NWs were collected by washing with DI water three times in centrifugation and redispersed in a 5 wt% PVP 55k solution for further synthesis. In the second step, 5 mL of smooth Au‐coated Ag NW stock dispersion from the first step was used, to which 1 mL 50 mM sodium citrate and 2.636 mL 5 wt% PVP 55k solution were added, after which 3.875 mL DI water with 4.24 µL HAuCl_4_ precursor solution was added. The bottle was heated in a 60 °C water bath for 3 h under stirring at 600 rpm and the sample color turned from goldish to completely gray. For investigating other additives, sodium citrate was replaced with 1 mL of 50 mM of sodium ascorbate (Asc), L‐arginine (Arg) or gamma‐aminobutyric acid (GABA), keeping the solution at the same pH by addition of 1 M NaOH solution. After finishing the synthesis, the sample was cooled under tap water, then washed with DI water 3 times by centrifugation at 2000 rpm, and the porous NWs were redispersed in DI water for further characterization.

### 4.3 Electron Microscopy

SEM images were obtained on a Zeiss Sigma 500 equipped with the Bruker Quantax EDS detector. The samples were prepared by filtering and depositing materials onto PVDF membranes, then rinsed with DI water and dried overnight at room temperature. Cross‐sectional images were obtained by placing cut samples on a 45° inclined aluminum stage. HAADF images were obtained using the Linköping FEI Titan^3^ 60–300 STEM, operated at 300 kV and equipped with a Super‐X EDX spectrometer. The samples were prepared by drop‐casting nanomaterial dispersions onto lacey carbon‐supported copper grids, dried, and cleaned with a vacuum cleaning instrument. A line profile of elemental analysis of the cross‐section on NWs was done by EDX. UV–vis absorption was measured with a Lambda 900 PerkinElmer absorption spectrometer. XRD spectra were measured by PANalytical Xpert operating at 45 kV, 40 mA. The samples were prepared in a similar way by depositing NWs on PVDF membranes as described for SEM samples. Zeta potentials were measured with folded capillary zeta cells by Zetasizer Nano ZS90. The samples were washed and redispersed in DI water.

### 4.4 Raman Spectroscopy

Raman and SERS experiments were performed using a Nikon Ti‐E inverted microscope equipped with a 60× air objective (Nikon CFI S Plan Fluor, 0.7 N.A), which offers a long working distance. The sample position was controlled by a Nikon TI‐S‐ER precision stage with a repeatability of less than 0.5 μm. A 532 nm laser with an output power of 10 mW illuminated the sample via a rear port of the microscope. The scattered light followed the same optical path but was redirected by a beamsplitter and guided through an optical fiber to an Andor Kymera 328i Raman spectrograph, equipped with a 600 lines/mm diffraction grating optimized (blazed) for 500 nm. Spectra were recorded with a thermoelectrically cooled EMCCD camera (Andor Newton DU970P‐BVF) set at −80 °C to enhance sensitivity. We collected the spectra using accumulation mode with a 1‐s exposure time and performed 30 or 60 accumulations, respectively. A 60‐s accumulation time was applied for measuring normal Raman spectra of the analytes (CV, MB, and MG). A 60‐s accumulation was also used for all MP measurements. All SERS measurements on analytes were performed with a 30‐s accumulation time. All obtained spectra were smoothed using a Savitzky–Golay filter (window size = 13, poly order = 3). Subsequently, baseline correction was applied using the asymmetric least‐squares (ALS) algorithm (iterations = 7, window size = 200, asymmetry parameter = 0.001).

PCA is a statistical approach that simplifies complex datasets by projecting them onto a new coordinate system defined by orthogonal vectors. These vectors, known as eigenvectors, are constructed as linear combinations of the original variables and represent the directions in which the data show the greatest variation. The new coordinate axes, called PCs, capture most of the variance in the dataset, with the first few components—often the first one to three—accounting for most of the information. In this work, PCA was employed to compress the large number of correlated wavenumbers into smaller, more manageable data sets. PCA was used to classify different analytes as well as different concentrations of these analytes on 3D‐AuNP‐Au@AgNW‐PDMS substrates in the spectral range of 1700–1000 cm^−1^. The score plot of the first two PCs was used to distinguish between the analytes and their concentrations.

For the SERS measurements, we faced two issues: the coffee‐ring effect and fluorescent BG. The diameters of the dried sample droplets were in the range of 5–6 mm (the droplet with analyte was normally slightly larger than the droplet with only 3D‐AuNP‐Au@AgNWs), as shown in Figure S14a,b, which also clearly show the coffee‐ring effect on the droplets. The fluorescent image (Figure S14d) shows strong fluorescence at the edges where dispersed matter has accumulated and which swamps the Raman signal. To address these problems, spectra were collected from the center to the edge of the droplet to balance fluorescence BG and analyte signal (Figure S15a–d). Based on these results, an inner circular region with a diameter of 2 mm, sharing the same center as the dried droplet, was selected as the measurement region. Because this region excludes the analyte‐enriched droplet edge, the local analyte concentration sampled for SERS may be lower than the nominal concentration used in the EF calculation.

### 4.5 3D‐AuNP‐Au@AgNWs on Glass Substrates

SERS substrates were prepared by drop‐casting 20 µL of a 3D‐AuNP‐Au@AgNW dispersion into round glass‐bottom well plates (9 mm diameter), followed by drying at 70 °C for 60 min on a shaker. After substrate preparation, 20 µL of analyte solutions at different concentrations (ranging from 1 × 10^−5^ to 1 × 10^−14^ M) were dropped onto the SERS substrates and dried at 70 °C for 30 min on a shaker to ensure sample consistency. During the analysis, three samples of each analyte were measured, and 15 spectra were collected for each analyte. The average spectra for each analyte are presented in Figure 3h, and band assignments are summarized in Table S1.

### 4.6 3D‐AuNP‐Au@AgNWs on Stretchable PDMS Substrates

Porous Au@Ag NW patches on PDMS substrates with nine 5 × 5 mm^2^ squares per sample were prepared in densities of 0.025, 0.05, and 0.1 mg/cm^2^ by filtration of dispersion through PVDF membranes (Figure S9). The membranes were dried on a 70° hot plate for 5 min. The patch size can easily be expanded to 3 × 3 cm^2^, as shown in Figure S10. Sylgard 184 was spin‐coated onto a glass wafer to form an 80 µm thick silicone elastomer film. The silicone film was semicured on a hotplate at 70° for 10 min, after which the PVDF membrane with the NWs was pressed on top and cured on the hotplate for another 10 min. The membrane was peeled off, leaving the NWs half‐embedded into the elastomer surface. After completely curing the PDMS, a stretchable substrate was obtained by peeling it off the glass support (Figure S10). The resulting device was conformal to different shapes of objects. To analyze the sensitivity of porous Au@Ag NWs‐PDMS SERS substrates, 20 µL of analyte solutions of three different concentrations (1 × 10^−5^, 5 × 10^−7^ and 1 × 10^−8^ M) were dropped onto the SERS substrates and dried. The average spectra from 20 measurements (4 samples with 5 random points from each sample) were obtained for each concentration (Figure S9a–c). To control the sensitivity of the SERS substrate, the same preparation was done for 1 × 10^−4^ M of each analyte on a clean PDMS substrate without NWs to obtain a normal Raman spectrum for each analyte.

### 4.7 MPs Preparation

To test the ability of MP detection by the 3D‐AuNP‐Au@AgNWs, we prepared polystyrene (PS), polypropylene (PP), polyethylene terephthalate (PET), and high‐density polyethylene (HD‐PE) MP debris from commercial products (PS from a food container, PP from goods packaging, PET from a water bottle, and HD‐PE from a milk container) in deionized water. This approach was intended to mimic MPs derived from waste and aged plastics in nature. To prepare the MPs, 1 g of each sample was grated and added to 20 mL of deionized water in a scintillation vial. The vials were then kept under UV light for 24 h. The mixtures were sonicated for 6 h in an ultrasonic bath, during sonication, the vials were vigorously shaken by hand several times. Large plastic particles were then removed, and the suspensions were filtered (Nylon net filter with 20 µm mesh). The remaining suspensions were centrifuged, and the collected MPs were used to prepare stock suspensions in deionized water at a concentration of 20 μg/mL. To prepare dried samples on glass, the suspension was diluted to 10 μg/mL, and 20 μL of this suspension was dried on a glass substrate. For the SERS sample preparation, 10 μL of the stock MP suspension was mixed with 10 μL of AuNW suspension to achieve a final concentration of 10 μg/mL of MPs. After 30 min, these suspensions were drop‐cast onto glass substrates.

### 4.8 Calculation of the Enhancement Factor

The enhancement factor (EF) of a SERS substrate is a crucial parameter in SERS sensing, and it can be calculated using the following equation [49, 50]:

(1) $$\mathrm{EF}=\frac{I_{\mathrm{SERS}}}{I_{R}}\times \frac{N_{R}}{N_{\mathrm{SERS}}}$$

where *I*_SERS_ and *I*_R_ represent the Raman signal intensities of the analyte with and without the SERS substrate, respectively. *N*_SERS_ and *N*_R_ refer to the number of analyte molecules in the SERS and regular Raman measurements, respectively. The number of molecules can be calculated as *N* = *C* × *V*, where *C* is concentration and *V* is volume. Since the films were prepared in glass‐bottom well plates with round 9 mm diameter wells and the laser spot area was kept consistent for all measurements (1.47 μm^2^), the equation can be simplified to:

(2) $$\mathrm{EF}=\frac{I_{\mathrm{SERS}}}{I_{R}}\;\times \;\frac{C_{R}}{C_{\mathrm{SERS}}}$$

where *C*_SERS_ and *C*_R_ represent the analyte concentrations in the SERS and normal Raman measurements, respectively. This simplified form provides a concentration‐based apparent enhancement factor for comparative purposes. Although useful for comparing analytical SERS performance, this approach does not explicitly account for the larger accessible surface area and volumetric hotspot distribution of the hierarchical 3D substrate. A geometry‐corrected EF was not reported because the effective active area could not be reliably quantified from SEM images. However, the dried droplets exhibit a coffee‐ring effect, resulting in a nonuniform analyte distribution, as shown in the Supporting Information. Since the SERS measurements were taken from the inner region of the droplet to avoid the strong fluorescence at the edge, the local analyte concentration in the measured area is likely lower than the nominal average concentration used in the calculation. Therefore, the reported EF values should be considered conservative apparent EF estimates. EF values for all analyte concentrations and characteristic peaks are presented in Figure S1, and the corresponding EF and LOD values are summarized in Table S2.

### 4.9 FDTD Simulations for Near‐Field Distribution

We used Ansys Lumerical FDTD software (https://www.ansys.com/products/optics/fdtd) to calculate near‐field (electrical) distributions around nanoparticles attached to a NW. The gold NW (diameter of 70 nm and length of 12 μm, with mesh size of 10 nm × 10 nm × 10 nm) was constructed as a cylinder model along the *X*‐axis, and gold nanospheres (with diameters of 10–20 nm, mesh sizes of 2 nm × 2 nm × 2 nm) were built for nanoparticles near the NW. We used PML boundaries in all directions and a TFSF source as the light source with propagation direction along the *Z*‐axis (forward from negative to positive *Z* coordinates). The near‐field distribution (near 532 nm) was acquired by a 2D power monitor in the *X* *Z*‐plane.

## Author Contributions

Y.L. and K.T. conceived of the project and contributed to the experimental design. Y.L. performed the materials synthesis and optical, scanning electron microscopy characterization. A.J., T.E., J.O.P. performed Raman experiments and data analysis. Y.L., Y.Y. performed the flexible substrate fabrication. D.L., M.J., Y.D. contributed to FDTD simulation. Y.L., J.P., P.P. performed scanning transmission electron microscopy characterization. K.T. guided the project. Y.L. and A.J. wrote the first draft of the manuscript, and all authors contributed to the finalization of the paper.

## Funding

This work was supported by the Swedish Research Council, European Research Council, Knut and Alice Wallenberg Foundation, Swedish Foundation for Strategic Research, and Swedish Government Strategic Research Area in Materials Science on Advanced Functional Materials at Linköping University.

## Supporting information

Supplementary Material

## Data Availability

The data that support the findings of this study are available from the corresponding author upon reasonable request. All raw data files are deposited in Zenodo data repository (https://doi.org/10.5281/zenodo.21905862).
