# Supplementary material for: Interfacial Engineering of Dual‐Layer Au‐Shelled Ag Nanowires: Decoupling Chemical Stability From Ultrasensitive Surface‐Enhanced Raman Spectroscopy Activity
Source: Small Sci. 2026 Sep 16;6(9):e70401. doi: 10.1002/smsc.70401 (PMC13587937; doi:10.1002/smsc.70401)
Supplement: Supplementary file 1 [file SMSC-6-e70401-s001.pdf]

Supplementary information

Interfacial Engineering of Dual-Layer Au-Shelled Ag Nanowires: Decoupling Chemical Stability from Ultrasensitive Surface-Enhanced Raman Spectroscopy Activity

Yuyang Li,[a#*] Ario Jafari,[b#] Dongqing Lin,[a] Justinas Palisaitis,[c] Yangpeiqi Yi,[a] Jonas Oshaug Pedersen,[b] Yulong Duan,[a] Per O.Å. Persson,[c] Magnus P. Jonsson,[a] Thomas Ederth,[b] Klas Tybrandt.[a*]

a) Laboratory of Organic Electronics, Department of Science and Technology, Linköping University, SE-60174 Norrköping, Sweden.

b) Division of Biophysics and Bioengineering, Department of Physics, Chemistry and Biology, Linköping University, SE-58183 Linköping, Sweden

c) Division of Thin Film Physics, Department of Physics, Chemistry and Biology, Linköping University, SE-58183 Linköping, Sweden

Tables S1-7

Figures S1-18

References

Table S1. Band assignments for crystal violet, malachite green, and methylene blue.

Band assignments	Wavenumber (cm^{-1})		
	Crystal violet[1,2,3,4]	Malachite green[1,2,5,6]	Methylene blue[7,8,9]
C-C ring stretch	1621, 1589, 1537, 1480, 1446, 1300	1615, 1592, 1488, 1447, 1294	1628
C-N stretch	1373	1398, 1368	1454 (asy), 1392 (sy)
C-H in-plane bending	1179, 1125, 988, 942, 916	1220, 1176, 987, 940, 916	1299, 1155, 1074, 1035, 953, 900
C-H out of plane bending	808, 763, 731	805, 760, 733	811, 774, 673
Skeletal deformation	566, 531, 445, 427	579, 530, 438, 428	598 (C-S-C), 503 (C-N-C), 451 (C-N-C)

Table S2. Analytical performance of the 3D-AuNP-Au@AgNW on glass SERS substrates for different analytes.

Analyte	Vibrational band	Detection limit	Enhancement Factor*
Crystal violet	C-C stretch (1623 cm^{-1})	1×10^{-13} M	9.2×10^{7}
	C-N stretch (1373 cm^{-1})	1×10^{-13} M	6.2×10^{7}
	C-H bending (1180 cm^{-1})	1×10^{-13} M	8.2×10^{7}
Malachite green	C-C stretch (1616 cm^{-1})	1×10^{-14} M	9.1×10^{8}
	C-N stretch (1368 cm^{-1})	1×10^{-13} M	1.4×10^{8}
	C-H bending (1173 cm^{-1})	1×10^{-13} M	2.0×10^{8}
Methylene blue	C-C stretch (1628 cm^{-1})	1×10^{-11} M	8.8×10^{5}

* Enhancement factors were calculated from nominal analyte concentrations at the lowest detectable concentrations and should be interpreted as concentration-based apparent EF values.

Table S3. Comparison of obtained SERS limits of detection (LOD) sensitivities on MG, CV, and MB molecules, with various 0D Au nanomaterial-based substrates in the literature.

**SERS substrates**	**MG**	**CV**	**MB**	**References**
AuNPs on Cu_2O microspheres	-	-	3.4×10^{-12}M	[10]
AuNRs with Electroextraction	-	-	1.5×10^{-10}M	[11]
Bimetallic Au/Ag Nanostars	-	-	1×10^{-8}M	[12]
Ag/Au nanocomposite particles	-	1×10^{-12}M	-	[13]
AuAg nanostars	-	1.5×10^{-11}M	-	[14]
AuNPs	-	1×10^{-9}M	-	[15]
Ag coated urchin-like Au NPs	2.5×10^{-9}M	-	-	[16]
Au nanorods in hydrogels	1.18×10^{-13}M		-	[17]
Au NBPs@Ag nanorods modified PDMS	1×10^{-10}M	-	-	[18]
This work	1×10^{-14} M	1×10^{-13} M	1×10^{-11} M	

Table S4. Fit parameters of the log–log Langmuir–Freundlich fits of SERS intensity vs concentration for CV, MG, and MB on Glass and PDMS. R^2 and *RMSE* (log-scale) quantify fit quality; I_{max} (counts) is the saturation intensity; K (M^{-1}) is the affinity constant; n is the heterogeneity exponent ($n < 1 \rightarrow$ heterogeneous sites); b is the background.

**Analyte**	**Sample**	R^2	**RMSE**	I_{max}	K	n	b
CV	Glass	0.997	0.057	780883	235383	0.488	435
	PDMS	0.988	0.053	34417	48309493	0.482	549
MG	Glass	0.996	0.060	6150339	1409	0.365	238
	PDMS	0.978	0.099	36342	49450281	0.556	452
MG	Glass	0.996	0.038	401334	14359	0.380	181
	PDMS	0.977	0.085	26553	60282785	0.657	150

Table S5. SNR statistics for the characteristic Raman bands of MG, CV, and MB at different concentrations. Mean SNR, SD (standard deviation), RSD (relative standard deviation), 95% CI (confidence interval), and p-values (vs. SNR = 3) are reported. For MB, data were available only to 10^{-12} M.

log C	Conc.	MG					CV					MB				
		mean SNR	SD	RSD (%)	95% CI	p vs SNR=3	mean SNR	SD	RSD (%)	95% CI	p vs SNR=3	mean SNR	SD	RSD (%)	95% CI	p vs SNR=3
-5	1e-5 M	419.9	152.4	36.30	419.9 ± 84.42	<0.001	176.6	63.55	35.98	176.6 ± 35.19	<0.001	118.3	42.47	35.89	118.3 ± 23.52	<0.001
-6	1e-6 M	364.8	104.1	28.53	364.8 ± 57.64	<0.001	389.5	143.3	36.78	389.5 ± 79.34	<0.001	85.60	38.25	44.68	85.60 ± 21.18	<0.001
-7	1e-7 M	210.4	45.62	21.68	210.4 ± 25.26	<0.001	57.76	18.21	31.53	57.76 ± 10.09	<0.001	21.64	14.21	65.64	21.64 ± 7.866	<0.001
-8	1e-8 M	195.2	94.23	48.26	195.2 ± 52.18	<0.001	105.7	56.35	53.30	105.7 ± 31.21	<0.001	34.90	21.55	61.77	34.90 ± 11.94	<0.001
-9	1e-9 M	182.2	53.80	29.54	182.2 ± 29.79	<0.001	33.52	24.24	72.30	33.52 ± 13.42	<0.001	58.07	18.20	31.35	58.07 ± 10.08	<0.001
-10	1e-10 M	309.7	270.2	87.26	309.7 ± 149.6	<0.001	51.70	30.31	58.63	51.70 ± 16.79	<0.001	36.02	14.54	40.38	36.02 ± 8.053	<0.001
-11	1e-11 M	85.48	56.30	65.87	85.48 ± 31.18	<0.001	30.89	22.61	73.22	30.89 ± 12.52	<0.001	20.61	12.29	59.61	20.61 ± 6.803	<0.001
-12	1e-12 M	74.36	45.67	61.41	74.36 ± 25.29	<0.001	46.23	34.02	73.57	46.23 ± 18.84	<0.001	1.790	0.550	30.73	1.790 ± 0.305	>0.999
-13	1e-13 M	14.75	8.797	59.65	14.75 ± 4.872	<0.001	47.06	32.80	69.69	47.06 ± 18.16	<0.001	—	—	—	—	—
-14	1e-14 M	9.841	5.019	51.00	9.841 ± 2.779	<0.001	2.519	0.216	8.563	2.519 ± 0.119	>0.999	—	—	—	—	—

Table S6. Statistical summary of microplastic SERS measurements from 25 spectra per polymer, including mean integrated area, SD, RSD, 95% CI, and Student's t-test p-values for selected characteristic bands.

Polymer	Peak (cm^{-1})	Mean integrated SERS area	SD	RSD (%)	95% CI	p-value
PS	1004	210.60	37.38	17.8	164.18–257.02	<0.001
PS	1602	35.00	5.99	17.1	27.56–42.44	<0.001
PS	3054	138.20	23.72	17.2	108.74–167.65	<0.001
PET	1097	229.83	56.14	24.4	160.12–299.52	<0.001
PET	1614	109.68	26.72	24.4	76.50–142.87	<0.001
PET	1729	54.11	11.37	21.0	39.98–68.234	<0.001
PP	973	25.54	13.09	51.2	11.81–39.28	0.002
PP	1458	22.70	11.25	49.5	10.90–34.50	0.002
PP	2880	131.47	61.91	47.1	66.51–196.44	0.002
PE	2848	3.46	1.23	27.1	2.78-4.14	0.002
PE	2873	7.46	2.06	34.7	6.32-8.60	0.002
PE	2920	2.98	0.98	39.71	2.44-3.55	0.002

Table S7. Characteristic Raman peaks for each polymer and their corresponding SERS-to-normal Raman area ratios, calculated after baseline correction using local integration ($\Delta = 10$ cm^{-1}).

	Peak position (cm^{-1}) and enhancement factor (a.u.)							
	**PE**		**PP**		**PET**		**PS**	
Peak 1	2848	6.9	973	9.9	1097	5.1	1004	39.2
Peak 2	2873	6.1	1458	8.4	1614	9.9	1602	13.4
Peak 3	2920	4.7	2880	9.5	1729	8.7	3054	21.4

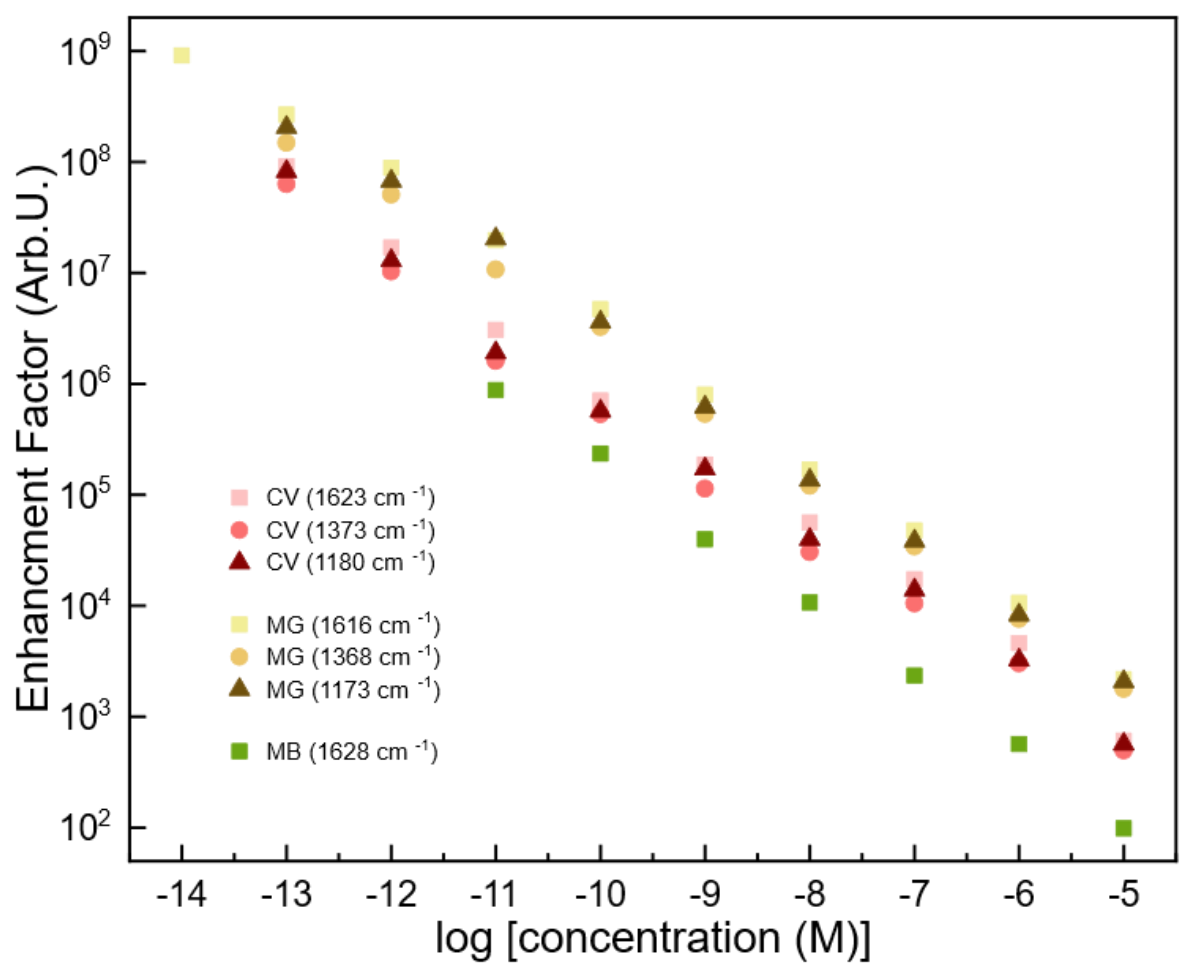

Figure S1. Raman enhancement factors for different bands of CV, MG, and MB, for different analyte concentrations.

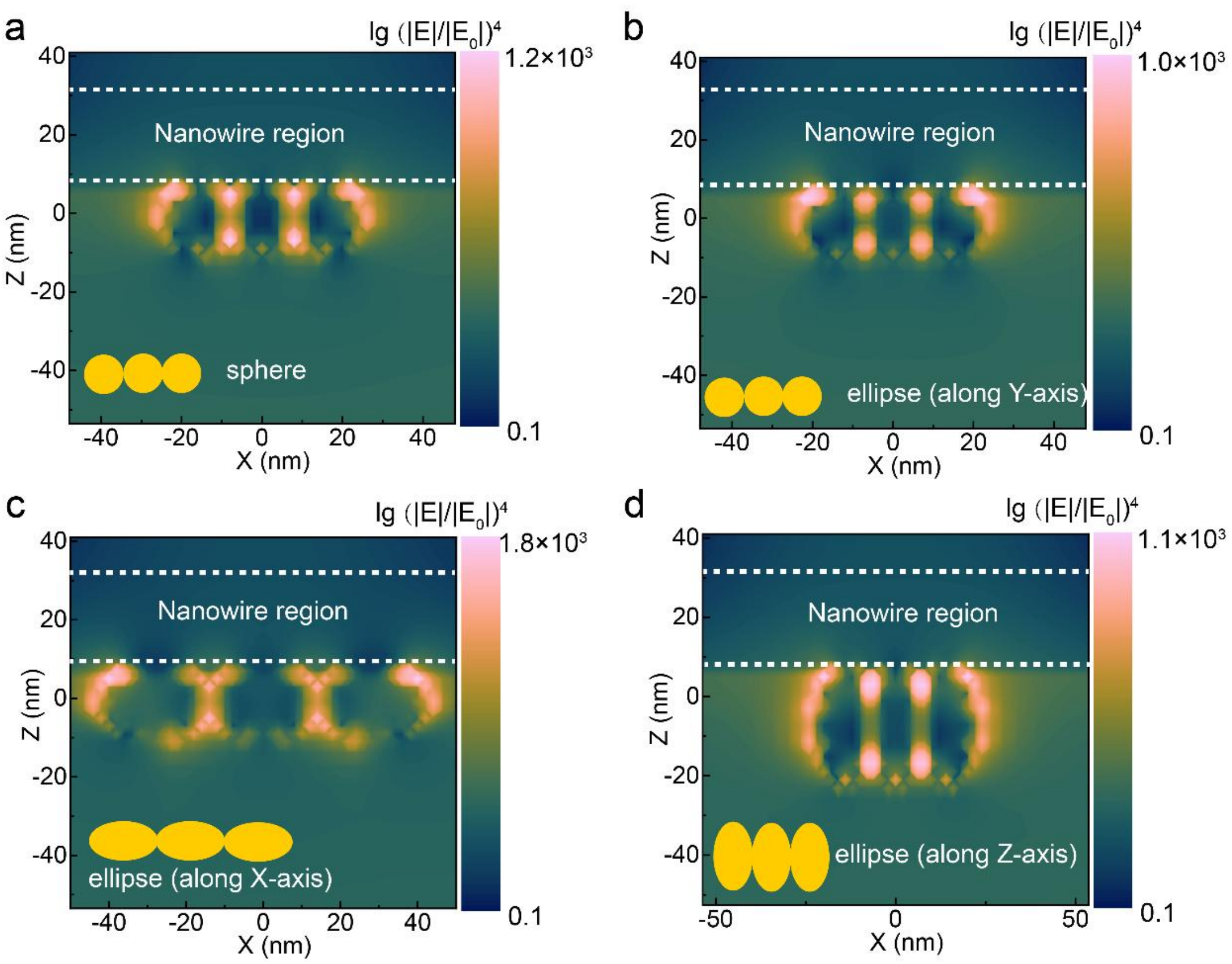

Figure S2. FDTD simulation results on the fundamental models of gold nanowires and gold nanoparticles with elliptic shapes. (a) perfect spherical shapes of nanoparticles for the reference model. (b) (c) (d) elliptic shapes of nanoparticles with long-axis along Y-axis, X-axis and Z-axis, respectively. For all elliptic nanoparticles, the long-axis and short -axis were set as 14 nm and 8 nm, respectively. The electrical polarization is along the X-axis. The wavelength of incident light is near 532 nm.

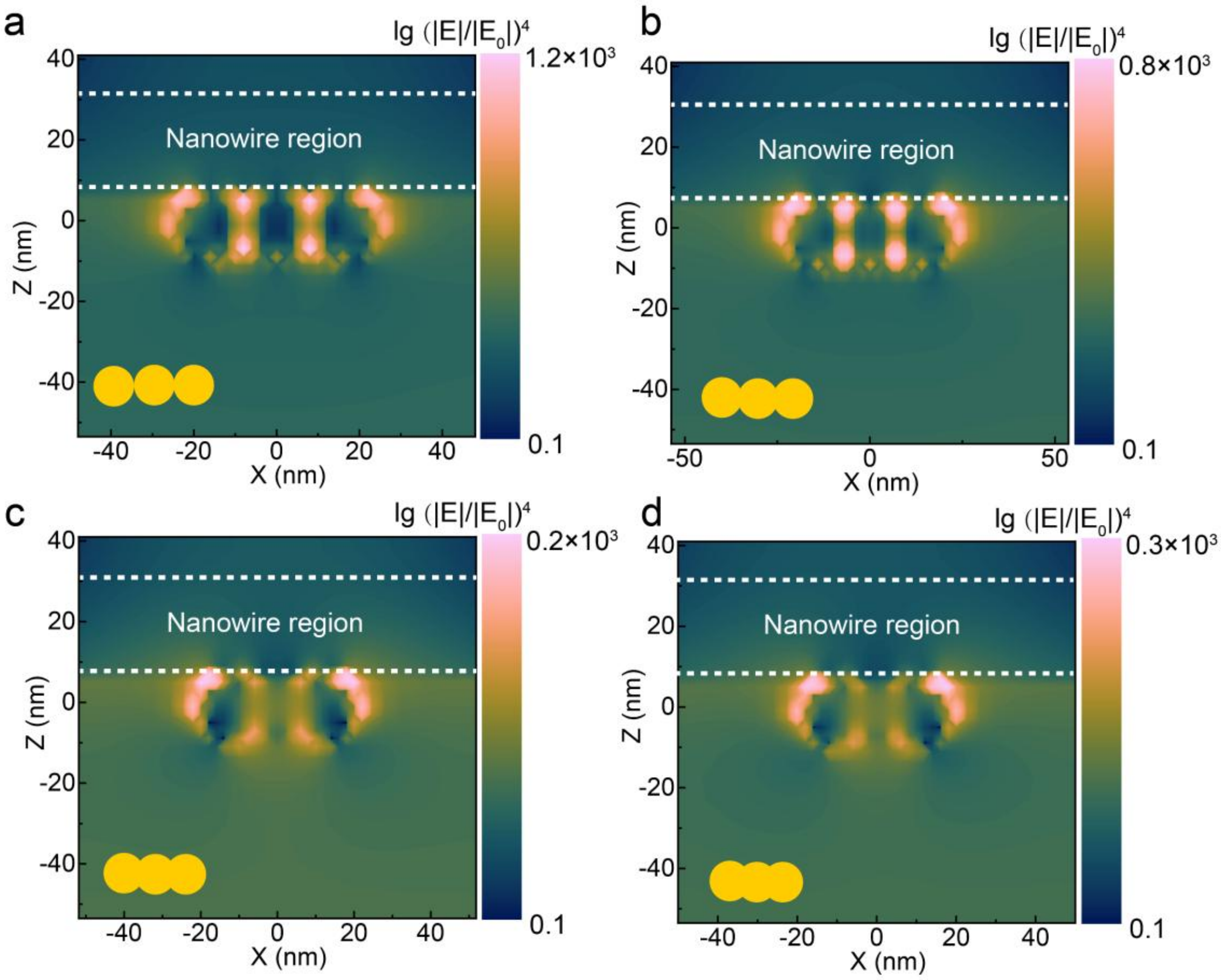

Figure S3. FDTD simulation results on the fundamental models of gold nanowires and gold nanoparticle aggregation with overlapped region. (a) perfect spherical shapes of nanoparticles for the reference model. (b) (c) (d) Nanoparticle aggregation with the overlapped length of 2 nm, 4 nm and 6 nm, respectively, along the X-axis. The electrical polarization is along the X-axis. The wavelength of incident light is near 532 nm.

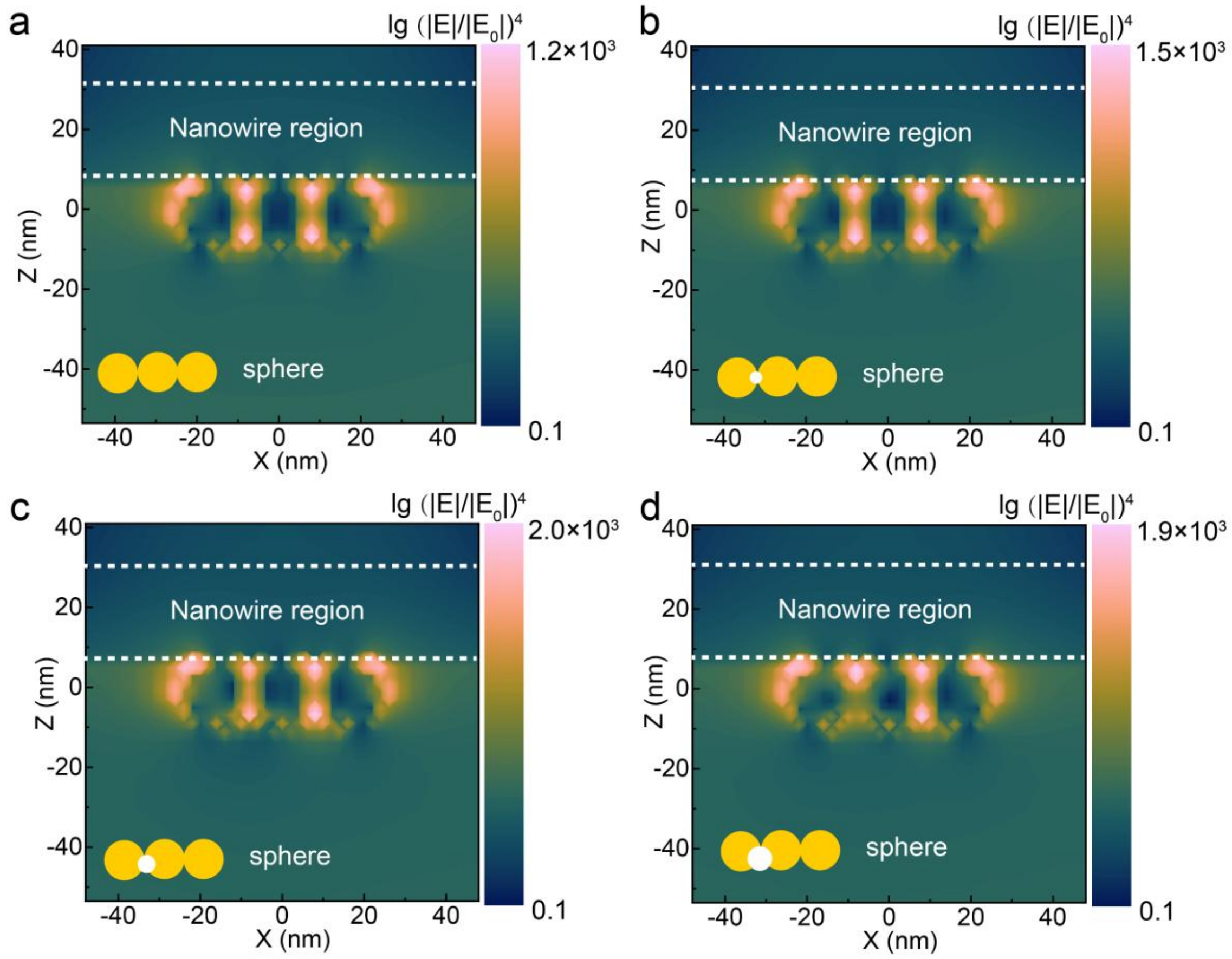

Figure S4. FDTD simulation results on the fundamental models of gold nanowires and gold nanoparticle aggregation with void defects (marked by a white sphere). (a) perfect spherical shapes of nanoparticles for the reference model. (b) (c) (d) the presence of void defects with sizes of 4 nm, 6 nm and 8 nm, respectively in the nanoparticle aggregation. The electrical polarization is along the X-axis. The wavelength of incident light is near 532 nm.

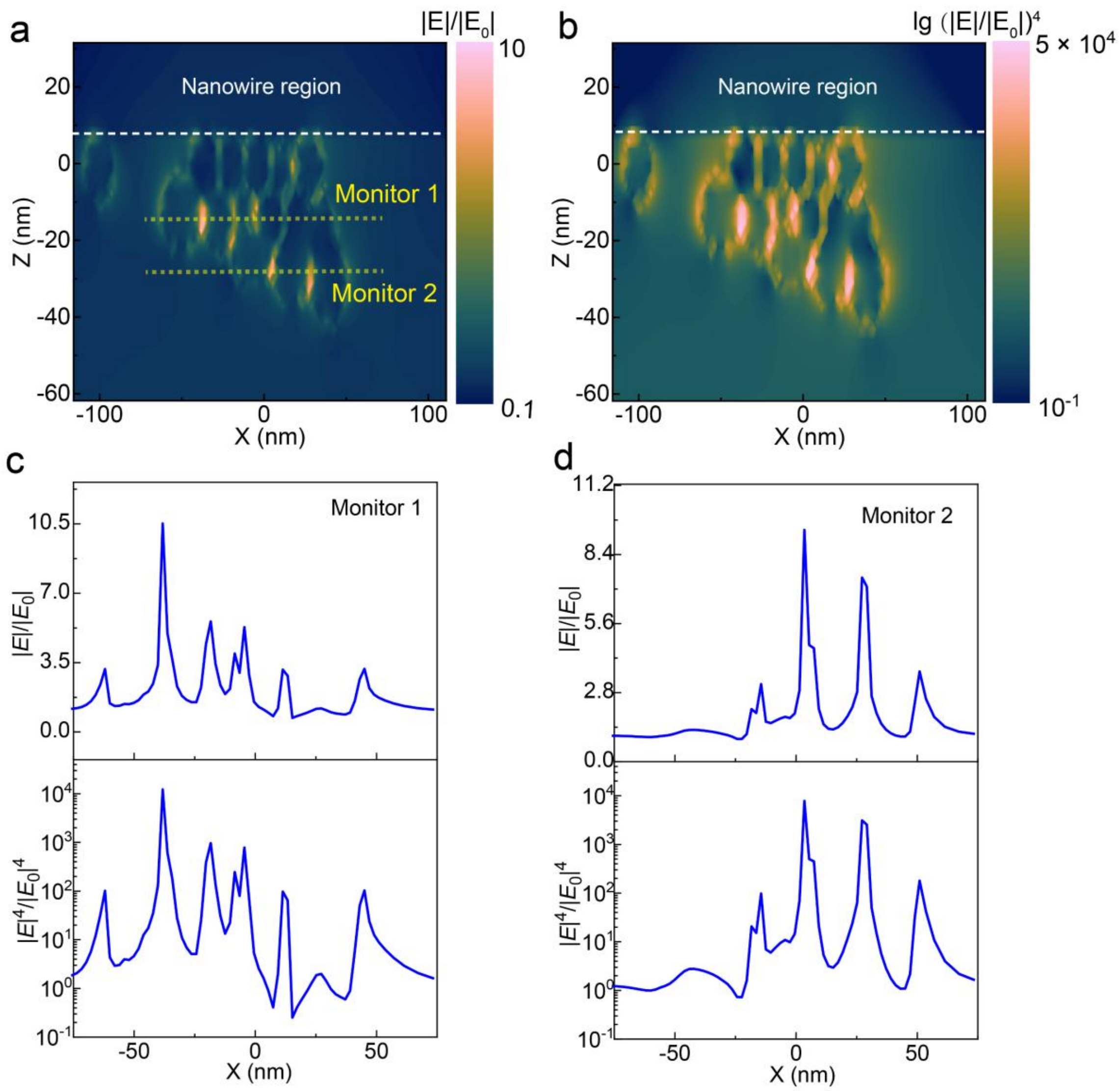

Figure S5. Near-field distribution (a) and enhancement factors (b) of gold nanoparticles and nanowire. Here, the nanoparticles are arranged in a random manner. The electric field dependent on the X coordination, detected from the linear Monitor 1 and 2, are shown in (c) and (d), respectively.

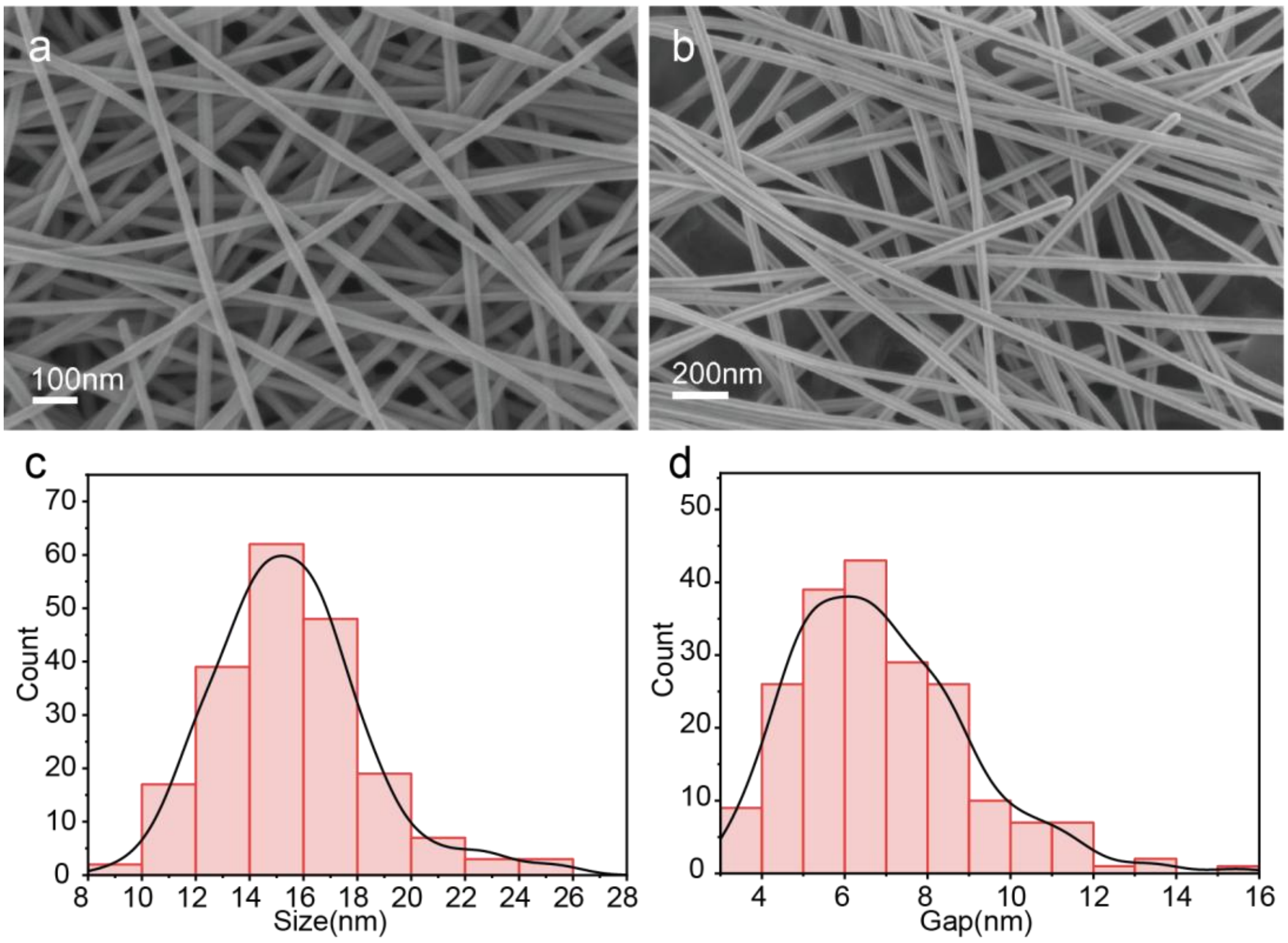

Figure S6. (a) Commercial Ag nanowires dispersed in water, with dimension of 12 μm in length and 20 nm in diameter. (b) Smooth Au-coated Ag bimetallic nanowires synthesized after the first step of the surface limited galvanic replacement reaction. Histogram of (c) size distribution and (d) gap distribution of porous Au-coated Ag nanowires as shown in Figure 1h-i, each measured with 200 data points by ImageJ for statistics.

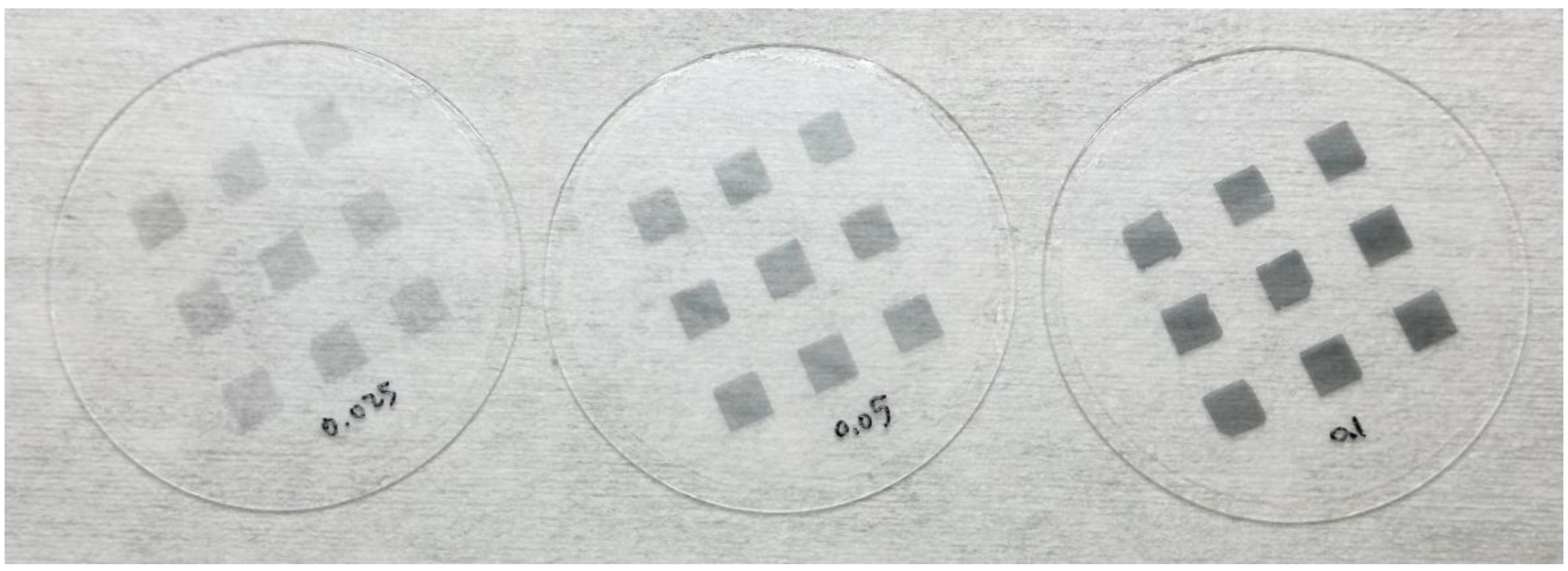

Figure S7. Porous Au NW PDMS (Sylgard 184 silicone elastomer) substrates on glass for SERS measurements, at 0.025mg/cm^3, 0.05mg/cm^3, and 0.1mg/cm^3, respectively.

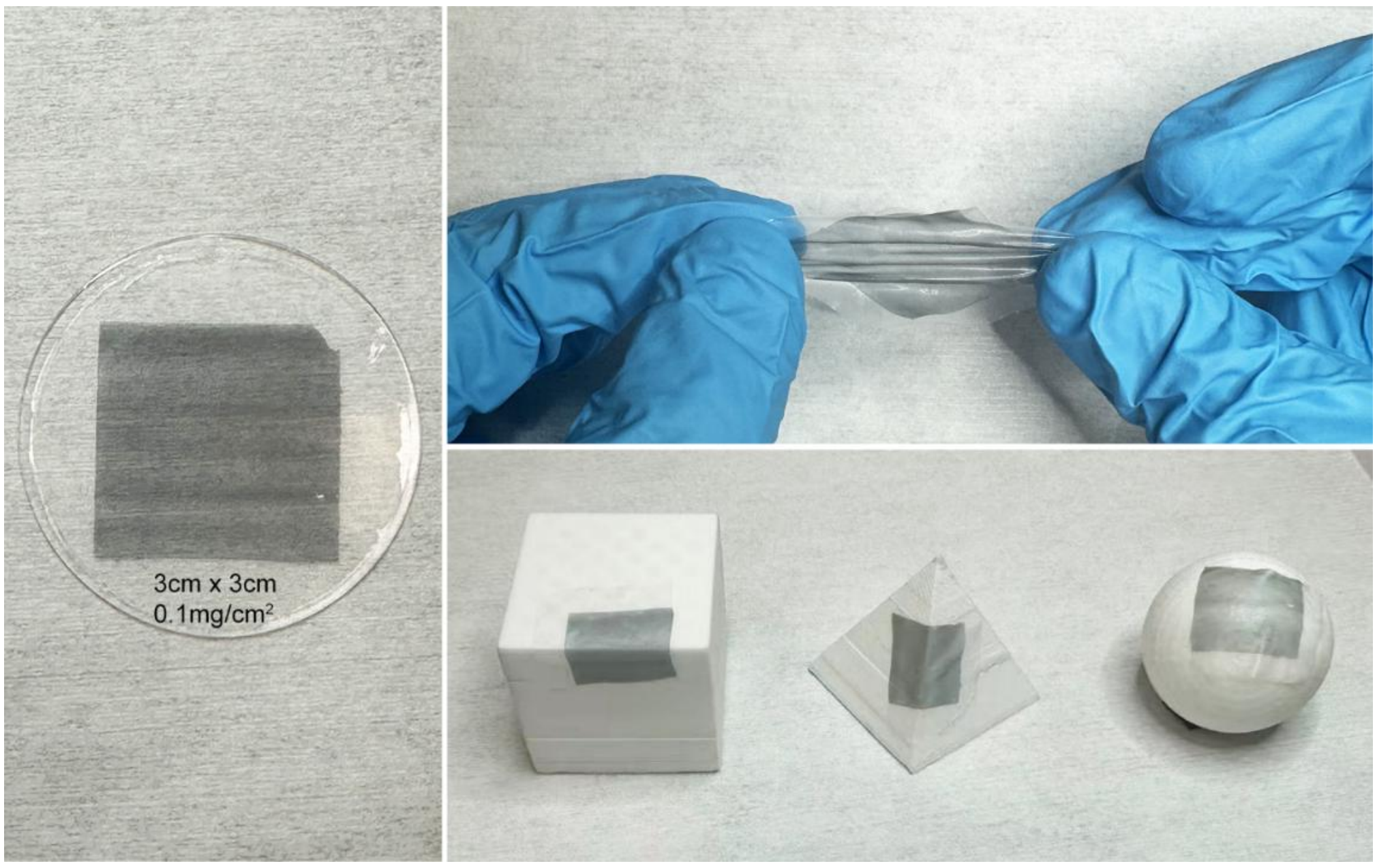

Figure S8. Soft stretchable AuAg-NW PDMS substrates. Left: large 3x3cm substrate with 0.1mg/cm^3 nanowire loading. Top right: After peeling off from the glass substrate. Bottom right: Conformal to different shapes of objects.

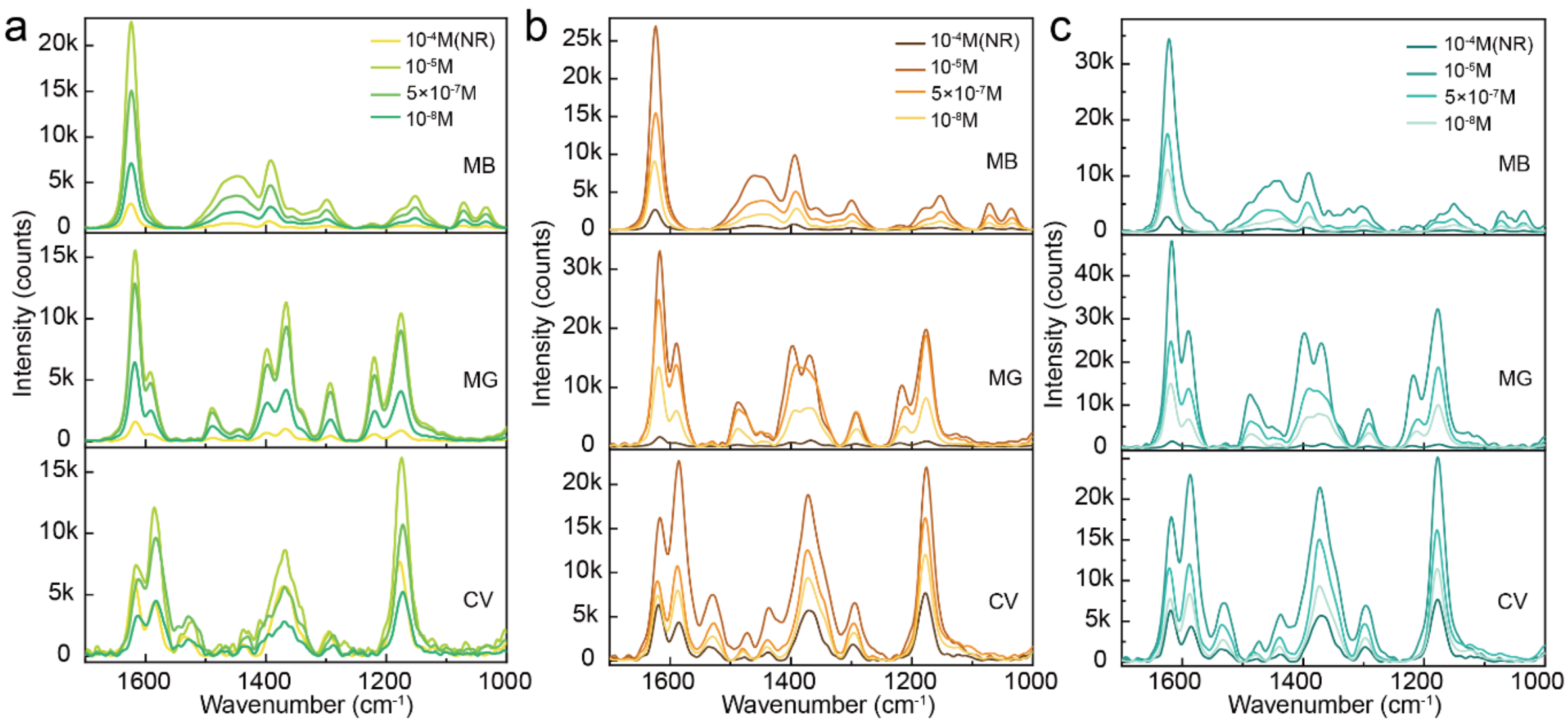

Figure S9. Normal Raman (NR) spectra of 10^{-4} M CV, MG, and MB, and SERS spectra of different concentrations of MB, CV, and MG measured on PDMS substrates (λ_0 = 532 nm, 10 mW, 30 s acquisition time) with different nanowire loadings: (a) 0.025 mg/cm^2, (b) 0.05 mg/cm^2, and (c) 0.1 mg/cm^2. Normal Raman spectra were recorded from the analytes on PDMS substrates without nanowires.

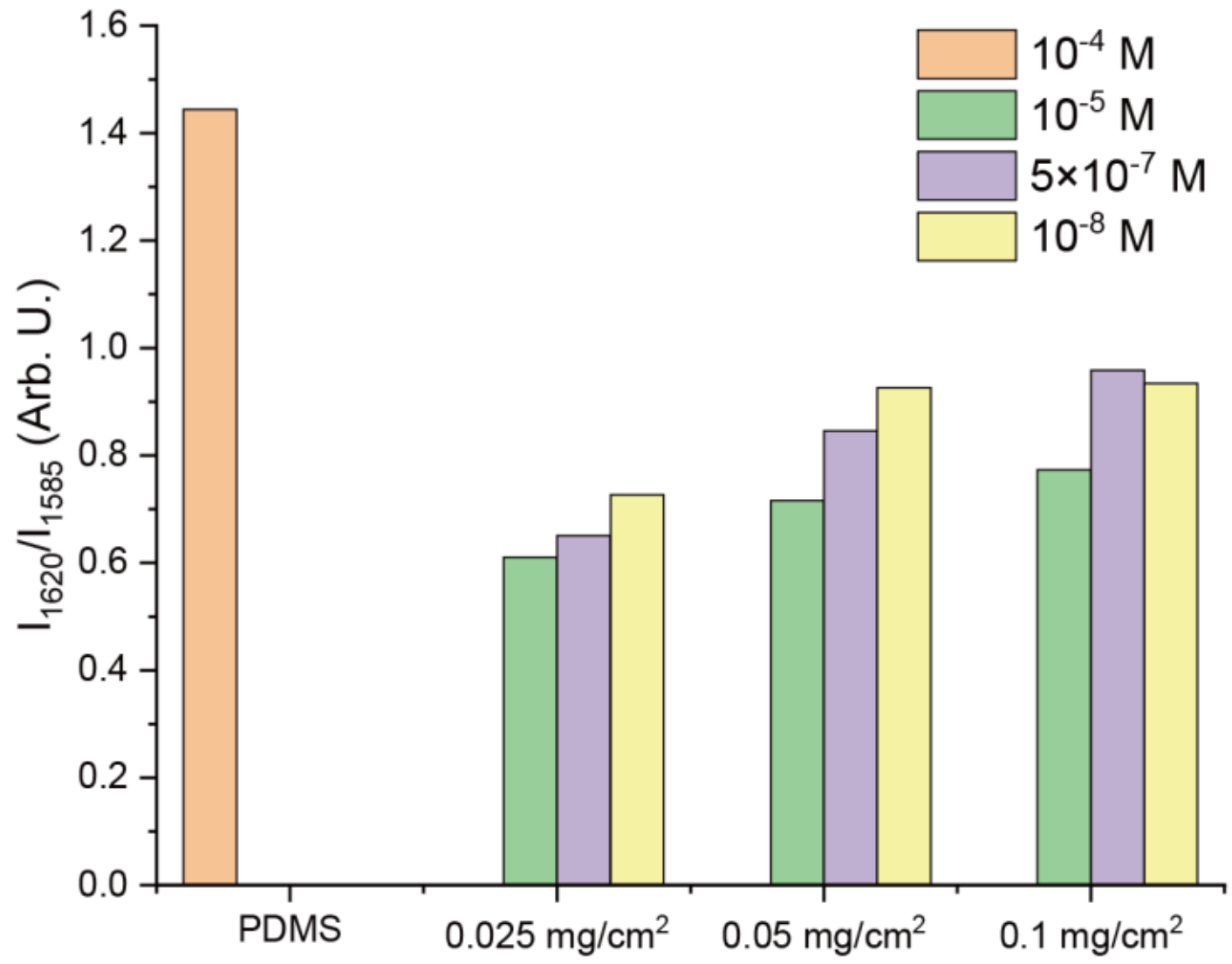

Figure S10. Intensity ratios of the peaks at 1620 cm^{-1} and 1579 cm^{-1}, respectively, for CV on PDMS and PDMS with different nanowire loadings, and at different CV concentrations.

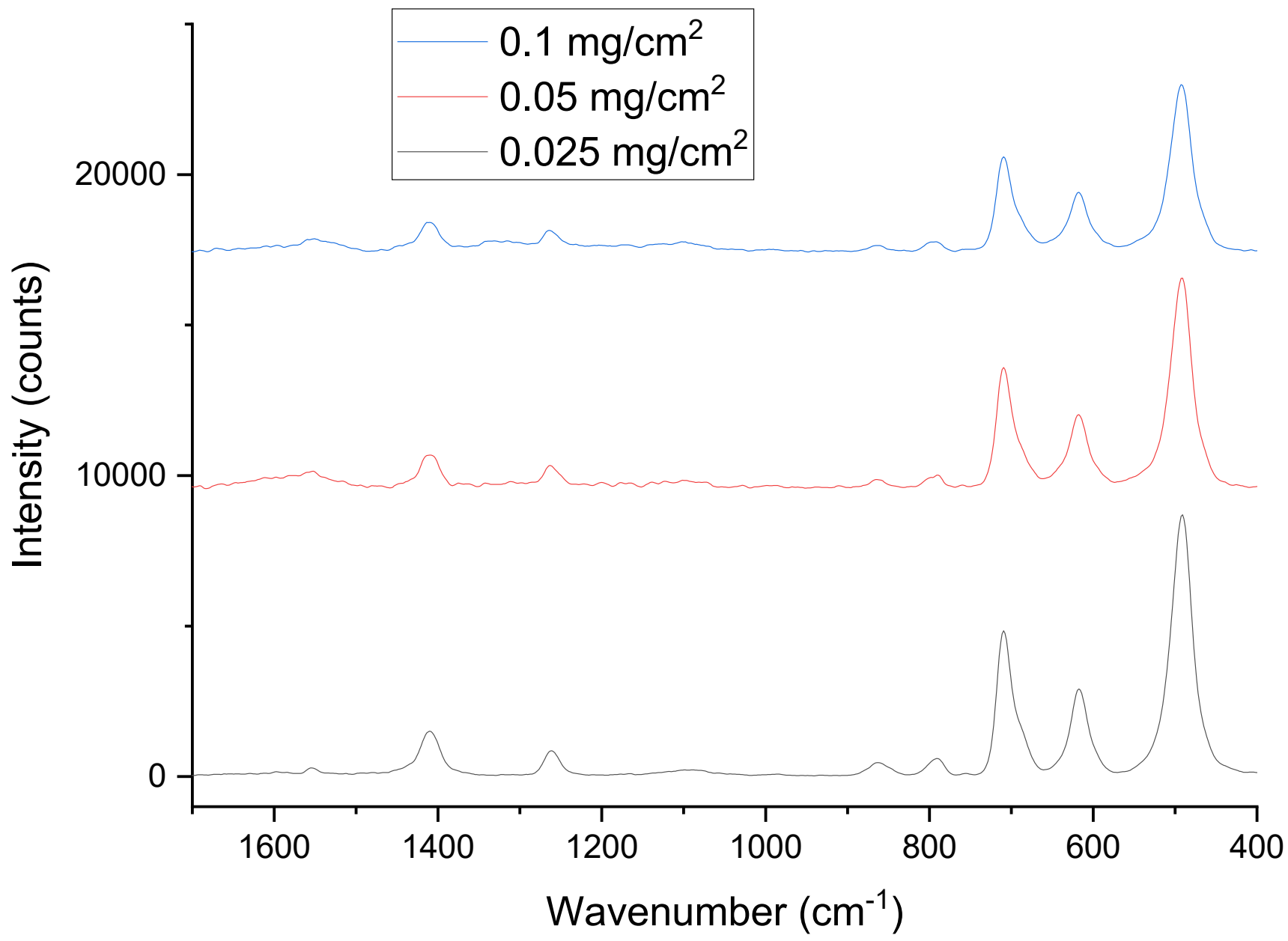

Figure S11. The Raman spectra of PDMS substrates with different nanowire loadings, but without any analyte. The spectra have been shifted vertically, for clarity.

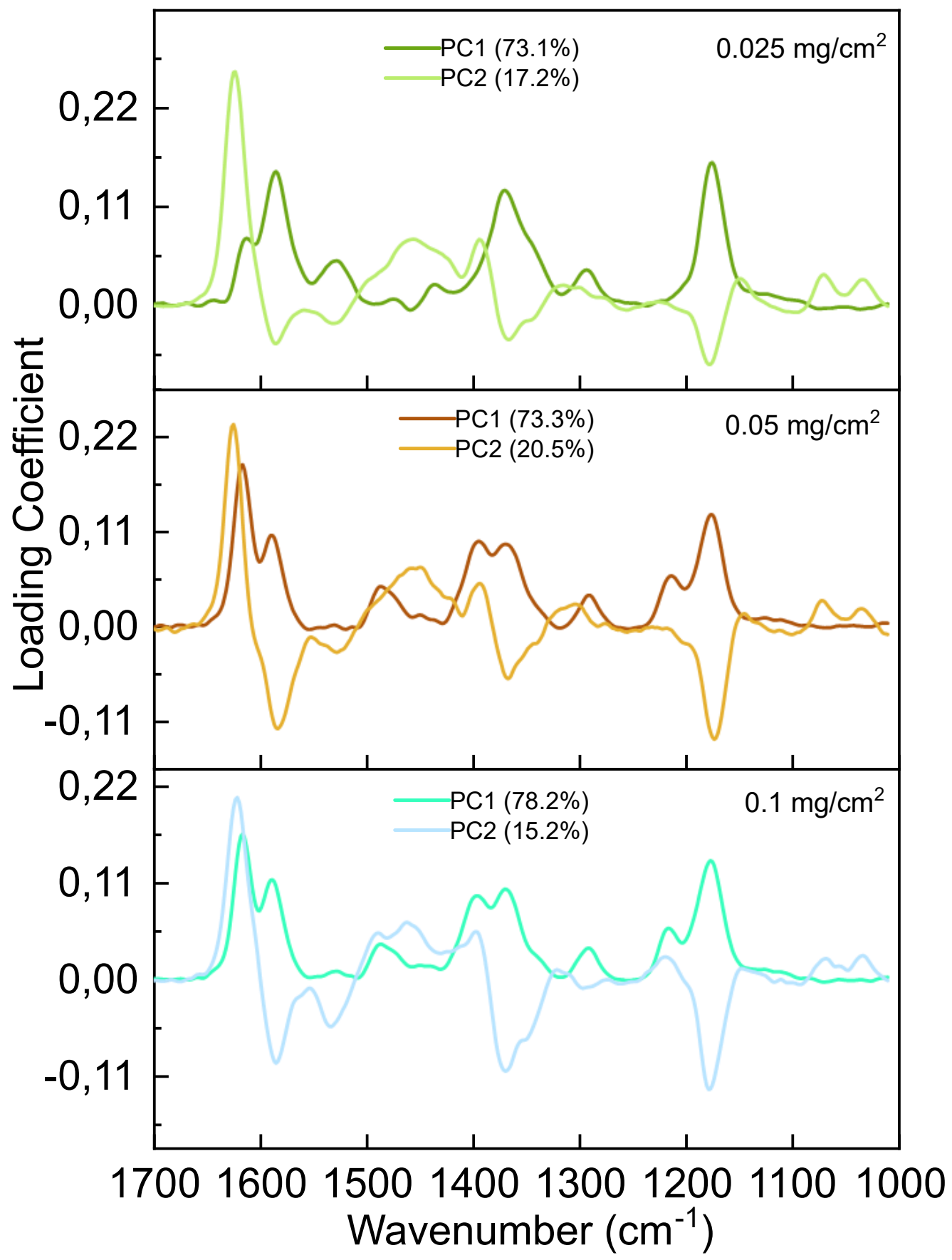

Figure S12. PCA loading plots of the contributions at different wavenumbers to the first two principal components (PC1 and PC2).

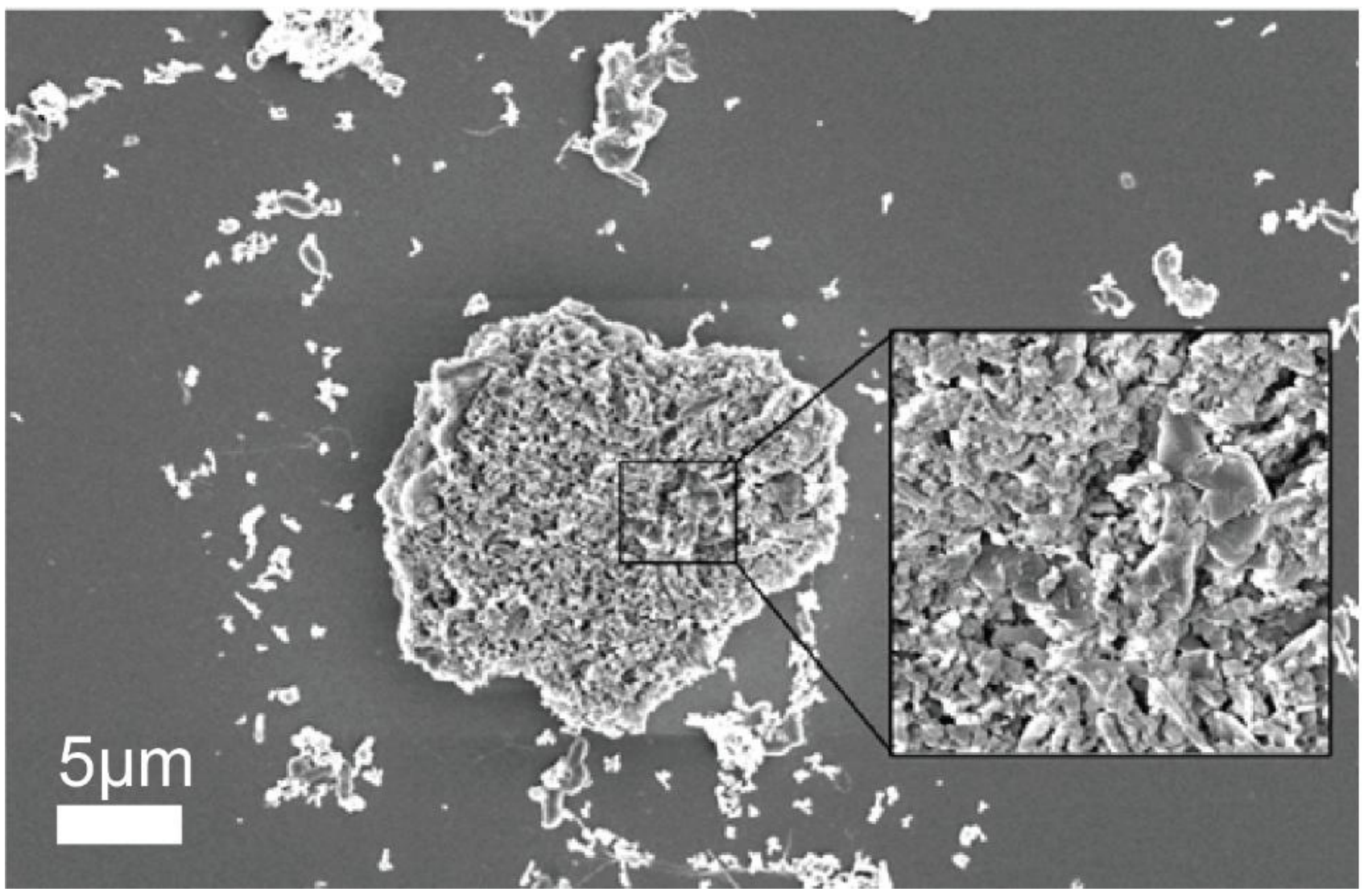

Figure S13. SEM images of aggregated PS microplastics.

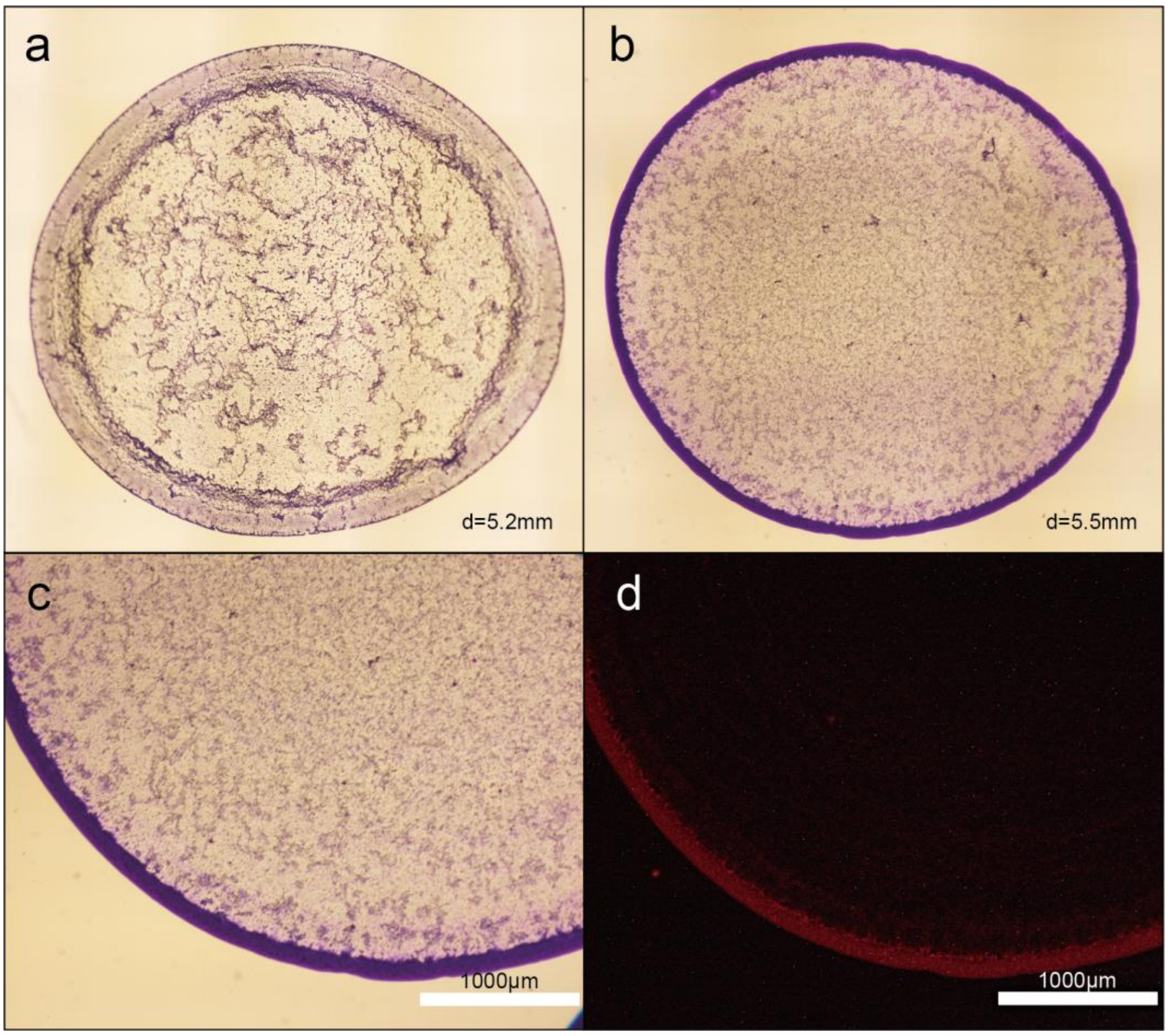

Figure S14. Optical images of (a) a dried NW droplet and (b) a dried droplet of NWs with CV. (c) Optical image and (d) fluorescent image, respectively, of the ring region of the dried droplet of NWs with CV. Scale bars in (c) and (d) are 1 mm.

Selection of droplet region for the measurements

To measure the average sizes of the dried droplets, we took optical images of the dried NWs (Figure S12a) and of dried NW samples with 10^{-6} M CV added before drying (Figure S12b). The images show that the size varies between 5–6 mm (the droplets with analyte are normally slightly larger than the droplets with only NWs). Optical images also clearly show the coffee-ring effect, which indicates that a high amount of analyte migrate to the droplet edge during drying. As the Raman spectra show a high fluorescence background in the edge region (discussed later), we also took fluorescent images of both droplets. The NW droplet does not show any fluorescences (not shown), but for the sample with analyte (CV) we can clearly see fluorescence from the ring, which fades when moving toward the center (see Figure S12c for the optical image and Figure S12d for the fluorescence image of the same region). This fluorescence background appears also in the Raman spectra, where it tends to swamp the Raman signal.

To find the best region for capturing spectral data, we collected spectra along a line from the center to the circumference ("coffee ring") of the dried droplet, using 21 points (the 1st point at the center and the 21st point at the circumference). To reduce the strong accumulation of fluorescent background, we used a laser power of 1 mW (while in the other measurements we used 10 mW). Figure S13a shows a Raman spectral map of the raw spectra from the center ($R/R_0 = 0$) to the circumference ($R/R_0 = 1$) of the droplet, where R_0 is the radius of the dried

droplet. This map indicates a strong fluorescent background in the ring region, as well as in areas near the ring. Raw Raman spectra from the center and from the ring are compared in Figure S13b. A possible explanation for the non-synchronous variation of the Raman signal with the changes in fluorescence is that the relative increase in the background from the ring region originates from accumulation of analyte molecules that are not adsorbed onto NWs, thus contributing to the fluorescence background but not to the SERS signal. This background can dominate the whole spectrum and mask identifiable peaks of the analyte. To evaluate where the background effect is lower, we integrated the entire spectral range for all spectra, shown in Figure S13c. This figure demonstrates that the fluorescence background is relatively constant from the center to the midpoint of the radius ($R = 0.5R_0$, where R_0 is the droplet radius).

To better focus on the identifiable peaks of the analyte (CV), we cut the spectra to the 1700–400 cm^{-1} range and applied baseline correction for comparison. Figure S13d shows the corrected spectra from the center, radial midpoint, and circumference. The results indicate that spectra from the center and midpoint are similar in intensity and signal-to-noise ratio, whereas spectra at the circumference (the “coffee-ring”) display weaker peaks and higher noise. This behavior can be attributed to the strong fluorescence background in the raw spectrum, which both covers analyte peaks and increases noise.

Based on this information, we selected the inner circular region of the droplet (within ~1 mm from the center) for data analysis, to achieve a better balance between peaks and background.

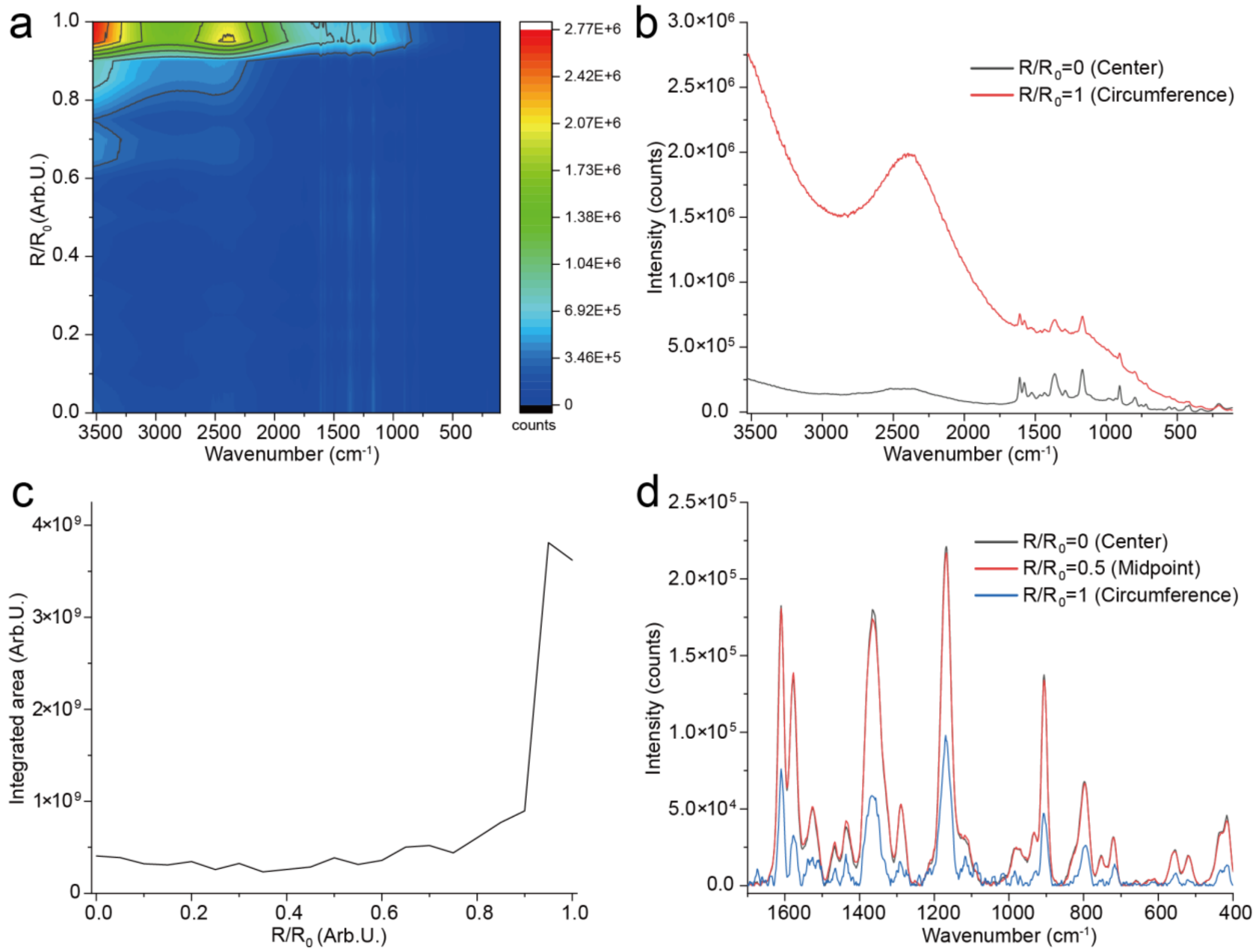

Figure S15. (a) Raman map of all raw spectra from the center ($R/R_0 = 0$) to the circumference ($R/R_0 = 1$) of the droplet, and (b) raw spectra obtained at the center and at the "coffee-ring" edge. (c) Integrated peak area over the whole spectrum for the same samples, and (d) baseline-corrected spectra at the center, radial midpoint, and circumference of the droplet, showing best residual Raman intensity at the radial midpoint.

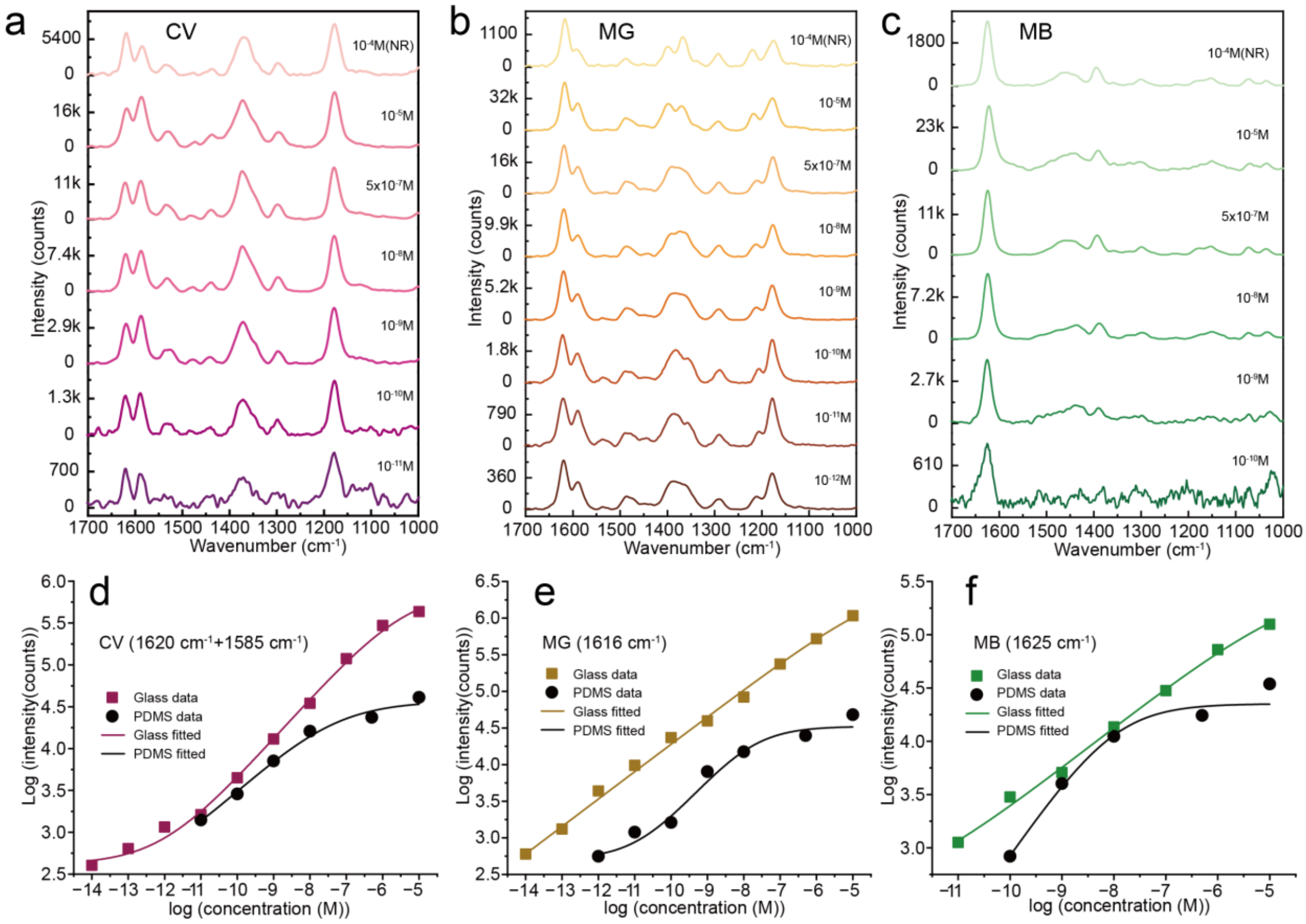

Figure S16. Raman spectra of different concentrations of (a) CV, (b) MG and (c) MB on the PDMS SERS substrates with 0.1 mg/cm^2 Au@Ag NW loading (λ_o = 532 nm, 10 mW, 30 s). SERS peak intensity maxima from PDMS SERS substrates (black data points and fitted lines) and 3D-AuNP-Au@AgNW on glass substrates at different added analyte concentrations, for (d) CV, (e) MG, and (f) MB.

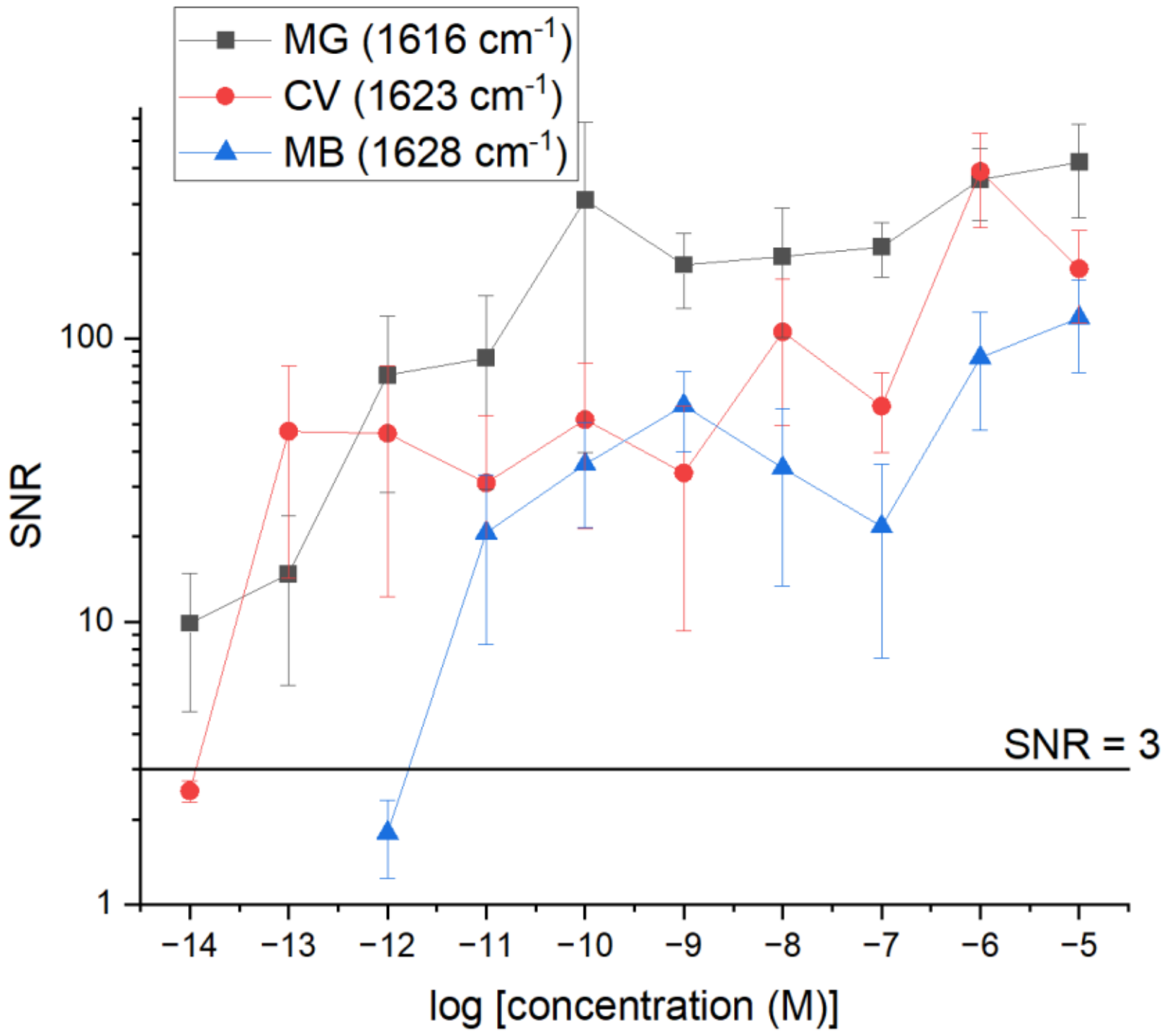

Figure S17. Signal-to-noise ratio (SNR) as a function of analyte concentration for malachite green (MG), crystal violet (CV), and methylene blue (MB), calculated from their characteristic Raman bands (1616, 1623, and 1628 cm^{-1}, respectively). Error bars represent mean ± SD from 15 spectra per concentration where replicate data were available. Full SNR statistics, including RSD, 95% CI, and p-values against the SNR = 3 threshold, are provided in Table S5. The horizontal line indicates the commonly accepted detection threshold (SNR = 3).

SNR was calculated as:

$$SNR = I_{peak}/\sigma_{noise}$$

where I_{peak} is the baseline-corrected peak intensity at the characteristic Raman band and σ_{noise} is the standard deviation of the signal in a nearby spectral region without Raman features. Baseline correction was performed using a linear fit to adjacent spectral windows. For MG and CV, SNR values were computed across 15 replicate spectra per concentration, while for MB a single-spectrum analysis was performed due to data availability.

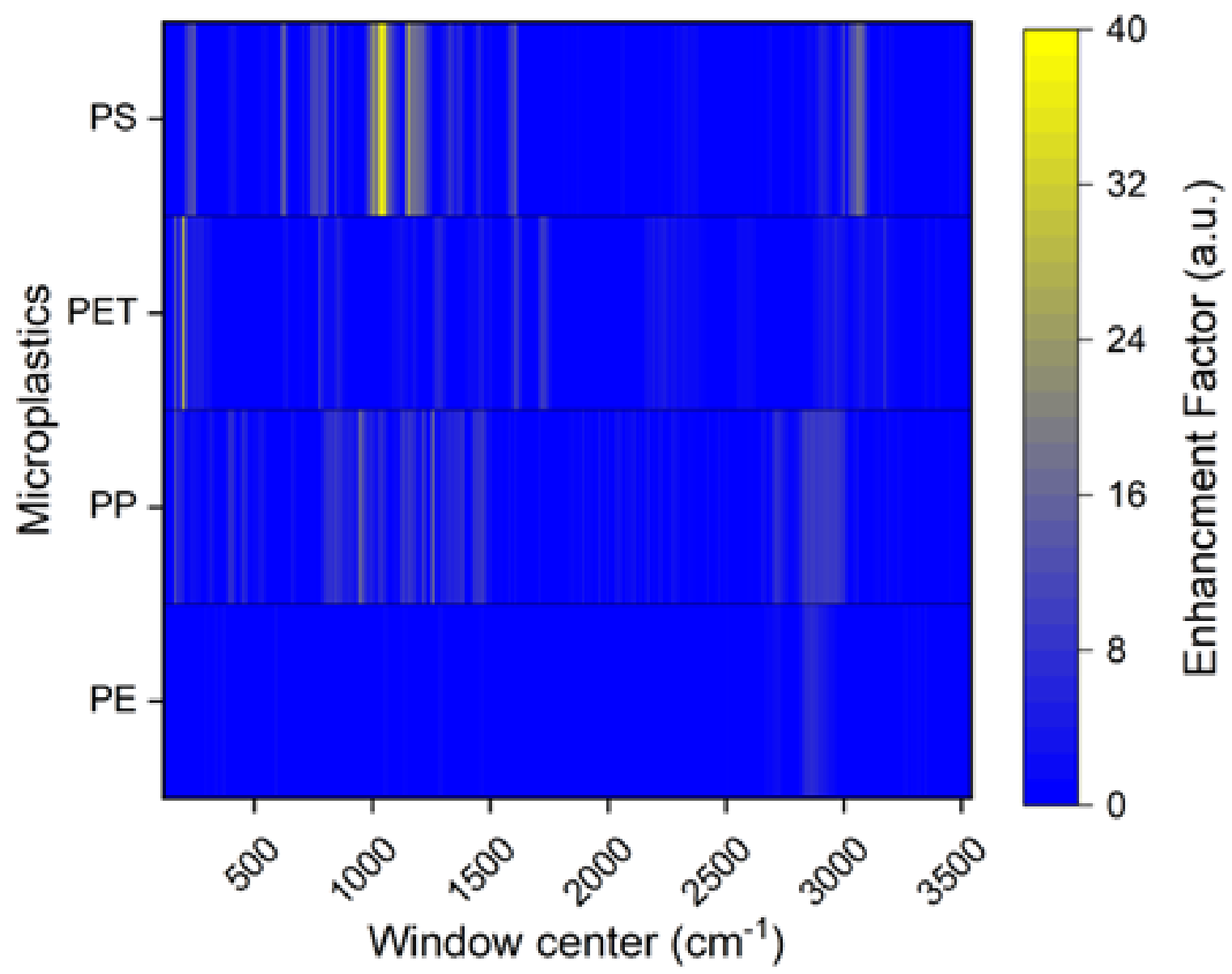

Figure S18. Heatmap showing that SERS enhancement occurs across multiple spectral regions for each polymer, not just a single peak.

References

1. Chen, X., Nguyen, T. H. D., Gu, L. & Lin, M. Use of Standing Gold Nanorods for Detection of Malachite Green and Crystal Violet in Fish by SERS. *J. Food Sci.* **82**, 1640–1646 (2017).

2. Schneider, S., Brehm, G. & Freunscht, P. Comparison of Surface-Enhanced Raman and Hyper-Raman Spectra of the Triphenylmethane Dyes Crystal Violet and Malachite Green. *Phys. status solidi* **189**, 37–42 (1995).

3. Angeloni, L., Smulevich, G. & Marzocchi, M. P. Resonance Raman spectrum of crystal violet. *J. Raman Spectrosc.* **8**, 305–310 (1979).

4. Liang, E. J., Ye, X. L. & Kiefer, W. Surface-enhanced Raman spectroscopy of crystal violet in the presence of halide and halate ions with near-infrared wavelength excitation. *J. Phys. Chem. A* **101**, 7330–7335 (1997).

5. Cen, Q., He, Y., Xu, M., Wang, J. & Wang, Z. Wavelength dependent resonance Raman band intensity of broadband stimulated Raman spectroscopy of malachite green in ethanol. *J. Chem. Phys.* **142**, 114201 (2015).

6. Chen, M., Huang, Y., Miao, J., Fan, Y. & Lai, K. A highly sensitive surface-enhanced Raman scattering sensor with MIL-100(Fe)/Au composites for detection of malachite green in fish pond water. *Spectrochim. Acta - Part A Mol. Biomol. Spectrosc.* **292**, 122432 (2023).

7. Pham, T. T. H. *et al.* Enhance Raman scattering for probe methylene blue

molecules adsorbed on ZnO microstructures due to charge transfer processes. *Opt. Mater. (Amst).* **120**, 111460 (2021).

8. Morales-Méndez, J. G. *et al.* Surface Enhanced Raman Spectroscopy of methylene blue deposited on Ag nanostructured substrates prepared by pulsed laser deposition. *Vacuum* **207**, 111580 (2023).

9. Rodríguez-Torres, M. del P., Díaz-Torres, L. A. & Romero-Servin, S. Heparin assisted photochemical synthesis of gold nanoparticles and their performance as SERS substrates. *Int. J. Mol. Sci.* **15**, 19239–19252 (2014).

10. Ashok Kumar, E., Jiann Wang, T.- & Chang, Y.-H. Ultrasensitive SERS substrates based on Au nanoparticles photo-decorated on Cu2O microspheres for the detection of rhodamine B and methylene blue. *Appl. Surf. Sci.* **585**, 152696 (2022).

11. Miranda, T. P., Orlando, R. M., Fantini, C. & Almeida, M. R. Enhancing Analytical Sensitivity and Selectivity for Methylene Blue Determination in Water Samples by Using Multiphase Electroextraction Coupled with Optical Absorption Spectroscopy and Surface-Enhanced Raman Scattering. *ACS Omega* **9**, 32769–32776 (2024).

12. Atta, S. & Vo-Dinh, T. A hybrid plasmonic nanoprobe using polyvinylpyrrolidone-capped bimetallic silver–gold nanostars for highly sensitive and reproducible solution-based SERS sensing. *Analyst* **148**, 1786–1796 (2023).

13. Kitaw, S. L. *et al.* An advanced plasmonic bimetallic nanostar composite for ultra-sensitive SERS detection of crystal violet. *Nanoscale* **17**, 508–519 (2025).

14. Li, Y.-L. *et al.* Study on the roughen process of branches of AuAg nanostars for the improved surface-enhanced Raman scattering (SERS) to detect crystal violet in fish. *Sensors Actuators B Chem.* **390**, 133936 (2023).

15. Sun, Z. *et al.* A SERS-active capillary for direct molecular trace detection in liquids. *Nanoscale Adv.* **3**, 2617–2622 (2021).

16. Liu, Y. *et al.* Sensitive detection of malachite green in aquaculture water by surface enhanced Raman scattering using silver coated urchin-like gold nanoparticles. *Colloids Surfaces A Physicochem. Eng. Asp.* **668**, 131485 (2023).

17. Ruan, S. *et al.* Preparation of 3D flexible SERS substrates by mixing gold nanorods in hydrogels for the detection of malachite green and crystal violet. *Microchim. Acta* **191**, 205 (2024).

18. Niu, F. *et al.* Controllable growth of Au NBPs@Ag nanorods modified PDMS as SERS substrate for rapid detection of malachite green in aquatic products. *Microchem. J.* **201**, 110569 (2024).
